# Codelivery of TGFβ and Cox2 siRNA inhibits HCC by promoting T-cell penetration into the tumor and improves response to Immune Checkpoint Inhibitors

**DOI:** 10.1093/narcan/zcad059

**Published:** 2024-01-09

**Authors:** Wookhyun Kim, Zhou Ye, Vera Simonenko, Aashirwad Shahi, Asra Malikzay, Steven Z Long, John J Xu, Alan Lu, Jau-Hau Horng, Chang-Ru Wu, Pei-Jer Chen, Patrick Y Lu, David M Evans

**Affiliations:** Sirnaomics Inc., 20511 Seneca Meadows Parkway, Suite 200, Germantown, MD 20876, USA; Sirnaomics Inc., 20511 Seneca Meadows Parkway, Suite 200, Germantown, MD 20876, USA; Sirnaomics Inc., 20511 Seneca Meadows Parkway, Suite 200, Germantown, MD 20876, USA; Sirnaomics Inc., 20511 Seneca Meadows Parkway, Suite 200, Germantown, MD 20876, USA; Sirnaomics Inc., 20511 Seneca Meadows Parkway, Suite 200, Germantown, MD 20876, USA; Sirnaomics Inc., 20511 Seneca Meadows Parkway, Suite 200, Germantown, MD 20876, USA; Suzhou Sirnaomics Pharmaceuticals, Ltd., Biobay, Suzhou, China; Sirnaomics Inc., 20511 Seneca Meadows Parkway, Suite 200, Germantown, MD 20876, USA; National Taiwan University College of Medicine, No. 1, Section 1, Ren’ai Rd, Zhongzheng District, Taipei City 100, Taiwan; National Taiwan University College of Medicine, No. 1, Section 1, Ren’ai Rd, Zhongzheng District, Taipei City 100, Taiwan; National Taiwan University College of Medicine, No. 1, Section 1, Ren’ai Rd, Zhongzheng District, Taipei City 100, Taiwan; Sirnaomics Inc., 20511 Seneca Meadows Parkway, Suite 200, Germantown, MD 20876, USA; Sirnaomics Inc., 20511 Seneca Meadows Parkway, Suite 200, Germantown, MD 20876, USA

## Abstract

Upregulation of TGFβ and Cox2 in the tumor microenvironment results in blockade of T-cell penetration into the tumor. Without access to tumor antigens, the T-cell response will not benefit from administration of the immune checkpoint antibodies. We created an intravenous polypeptide nanoparticle that can deliver two siRNAs (silencing TGFβ and Cox2). Systemic administration in mice, bearing a syngeneic orthotopic hepatocellular carcinoma (HCC), delivers the siRNAs to various cells in the liver, and significantly reduces the tumor. At 2 mg/kg (BIW) the nanoparticle demonstrated a single agent action and induced tumor growth inhibition to undetectable levels after five doses. Reducing the siRNAs to 1mg/kg BIW demonstrated greater inhibition in the presence of PD-L1 mAbs. After only three doses BIW, we could still recover a smaller tumor and, in tumor sections, showed an increase in penetration of CD4+ and CD8+ T-cells deeper into the remaining tumor that was not evident in animals treated with non-silencing siRNA. The combination of TGFβ and Cox2 siRNA co-administered in a polypeptide nanoparticle can act as a novel therapeutic alone against HCC and may augment the activity of the immune checkpoint antibodies. Silencing TGFβ and Cox2 converts an immune excluded (cold) tumor into a T-cell inflamed (hot) tumor.

## Graphical Abstract

**Graphical Abstract:**
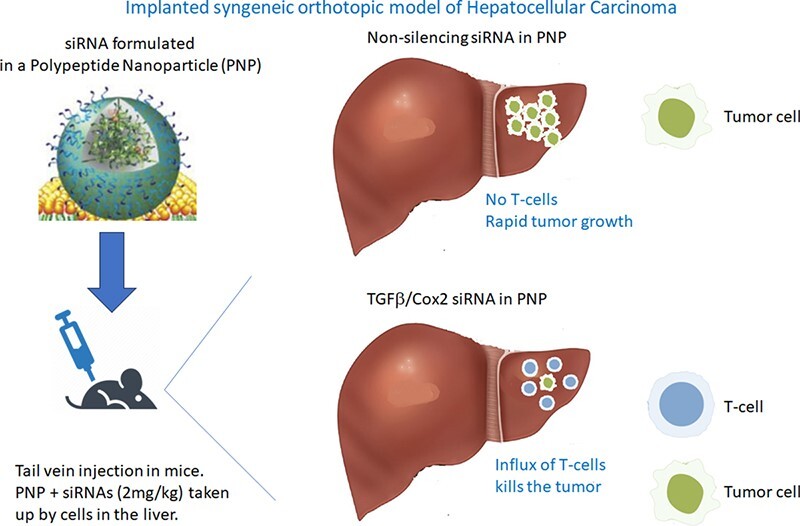

## Introduction

TGFβ is a pleiotropic agent that inhibits cancer at earlier time points and augments cancer growth rate at later stages of disease (1). Elevated TGFβ levels have been correlated with more aggressive cancer growth and poor prognosis for patients (2,3) and TGFβ in the tumor stroma can affect tumor growth rate (4). TGFβ plays a significant role in the etiology of cancer—including modulation of the immune environment (1,3–6)—and this seems particularly evident in Hepatocellular Carcinoma (1). Defective TGFβ responsiveness in immune cells can lead to chronic inflammation and the production of a pro-tumorigenic environment (7). A crucial role of TGFβ in T-cell regulation was suggested by the early discovery of the anti-proliferative effects of TGFβ on T cells *in vitro* (7). TGFβ inhibits the acquisition of most, if not all, effector functions by naive T cells: CD8+ T-cells activated in the presence of TGFβ do not acquire CTL function, and CD4+ T-cells fail to become TH1 or TH2 cells (7).

Cox2 also plays a critical role in altering immune response (8). Zelenay *et al.* (8) showed that the growth of tumors formed by mutant BrafV600E mouse melanoma cells in an immunocompetent host required the production of prostaglandin E2, which suppresses immunity and fuels tumor-promoting inflammation. Pre-clinical data demonstrated that inhibition of Cox2 synergizes with anti-PD-1 blockade in inducing eradication of tumors, implying that Cox2 inhibitors could be useful adjuvants for immune-based therapies in cancer patients (9). Furthermore, studies have shown that, within hepatocellular carcinoma (HCC), increased Cox2 production results in increased Treg infiltration (9). Consequently, we hypothesized that reducing Cox2 as well as TGFβ may be a good way to improve the immune environment around a tumor and promote therapeutic efficacy against a variety of tumors.

In colorectal cancer (CRC), in mice with progressive liver metastatic disease, blockade of TGFβ signaling rendered tumors susceptible to anti-PD-1/PD-L1 therapy (10). TGFβ inhibitors such as galunisertib, administered with anti-PDL1 antibodies, led to complete tumor rejection in metastatic CRC (10).

Galunisertib is a small molecule inhibitor of the TGFβ pathway that acts by inhibiting signaling through TGFβ receptor I (5). As a monotherapy, galunisertib has shown some antitumor activity in a variety of tumors, including durable and long-term responses in patients with glioma (5). In preclinical models, galunisertib demonstrated the ability to modulate antitumor T cell immunity, alone and in combination with PD-L1 checkpoint blockade (5). Mariathasan *et al.* showed that therapeutic co-administration of TGFβ-blocking galunisertib and anti-PD-L1 antibodies reduced TGFβ signaling in stromal cells, facilitated T-cell penetration into the center of tumors, and provoked vigorous anti-tumor immunity and tumor regression (6). Their data provided a strong rationale to clinically explore the potential of galunisertib to enhance anti-tumor immune response, particularly, in combinations with PD-L1/PD-1 checkpoint inhibitors, and also prompted this work, studying the effect of silencing the targets using siRNA.

We demonstrate that a formulation consisting of two siRNAs (targeting TGFβ and Cox2), incorporated in a polypeptide nanoparticle, can simultaneously deliver the 2 siRNAs to cells in the liver *in vivo* where they demonstrate gene silencing. The nanoparticle consists of the branched polypeptide composed of histidine and lysine—known as HKP or HKP(+H). These polypeptides have been used extensively to obtain systemic delivery of siRNAs to various tissues and tumors (11–18).

When delivered intravenously, the PNP delivers the two siRNAs to the liver. In a syngeneic orthotopic animal model of HCC, we demonstrated that silencing these two targets promotes single agent antitumor activity and, at lower doses, demonstrated that they can augment the activity of anti-PDL1 antibody in this model, possibly by increasing T-cell penetration into the tumor.

## Materials and methods

### Cell culture

The human hepatocellular carcinoma cell lines, HepG2, SNU-182, and murine hepatoma cell line Hepa1–6 were obtained from ATCC (Rockville, MD). Cells were cultured in standard media supplemented with 10% FBS; HepG2 in EMEM; SNU182 in RPMI and Hepa1–6 in DMEM medium. All media were obtained from ATCC. Human hepatic stellate cells were obtained from Zen-Bio inc. (Research Triangle Park, NC) and cultured in HSGM medium according to the Zen-Bio protocol.

### Quantitation of siRNA effects in *in vitro* systems

We designed 25mer siRNAs against both TGFβ and Cox2 and validated their ability to potently silence their respective genes *in vitro* using cells expressing the gene.

siRNA transfections were performed one day after seeding cells in 12-well plates at a density of 2.5 × 10^5^cells/well. siRNAs were transfected into cells using Lipofectamine RNAiMAX (Life Technologies, Carlsbad, CA) according to the manufacturer's recommendations. 24 h after transfection, total RNA was extracted using RNeasy Plus mini kit (QIAGEN), following the protocol suggested by the manufacturer. cDNA synthesis was performed using Maxima First Strand cDNA kit (Life Technologies).

RNA level was determined by TaqMan-qPCR (TaqPath PCR master mix). The sequences of primers for human TGFβ-1 were -5′ GCCTTTCCTGCTTCTCATGG-3′ (forward) and 5′-CGTGGAGTTTGTTATCTTTGCTG-3′ (reverse), for human HPRT- were 5′-GACTTTGCTTTCCTTGGTCAGG-3′ (forward) and 5′- AGTCTGGCTTATATCCAACACTTCG-3′ (reverse). Cox-2 expression level was quantified with TaqMan assay from ThermoFisher Scientific (Hs00153133_m1). Cox2 and TGFβ1 mRNA expression level in mouse Hepa1–6 cells was quantified with TaqMan assays from ThermoFisher Scientific—mCOX2 TaqMan assay ID: Mm00478374_m1, mTGFβ1 TaqMan assay ID: Mm01178820_m1 and mHPRT TaqMan assay ID: Mm03024075_m1. Amplification conditions were set at 50°C for 2 min, 95°C for 20 s, and included 40 cycles of 95°C for 15 s and 60°C for 1 min. mRNA level in all samples was calculated relative to RNA level of a non-treated control. HPRT was used as an internal standard for all samples.

### Cell viability assay

Human primary hepatic stellate cells (hHSC) were seeded in 96-well plates at a density of 1.2 × 10^3^ cells/well. The next day cells were transfected with Cell Death (CD) or non-silencing (NS) siRNAs formulated with HKP or Lipofectamine RNAiMAX (Life Technologies, Carlsbad, CA). Seventy-two hours after transfection, the number of viable cells was determined with CellTiter-Glo(R)-2.0 reagent (Promega, Madison, WI) by measuring luminescent signal using a Cytation 5 plate reader (BioTek Inc., Winooski, VT).

### Cellular uptake of PNP

Human primary hepatic stellate cells were transfected with AF647-labeled non-silencing (NS) siRNA formulated with HKP or Lipofectamine RNAiMAX (Life Technologies, Carlsbad, CA). Images were taken 24 h after transfection using a Cytation 5 imaging station (Biotek Inc., Winooski, VT).

### Optimizing formulation of nanoparticles

HKP stock was prepared in water and diluted to various concentrations. TGFβ and Cox2 siRNA stocks were prepared in water and mixed at TGFβ:Cox2 siRNA weight ratio of 1:1. Peptide nanoparticle (PNP) formulations were prepared using a microfluidic mixing system with staggered herringbone patterned mixer (Precision NanoSystems, Vancouver, BC, Canada). The siRNA and HKP were mixed at a volume ratio of 1:1 at a 10 ml/min total flow rate. Formulations were incubated for 30 min at room temperature. Particle size and zeta potential were determined by dynamic light scattering with Zetasizer Ultra (Malvern Panalytical, MA). A gel retardation assay was used to evaluate the efficiency of complex formation. Briefly, pre-formed PNPs were mixed with RNA loading dye and applied to 2% agarose gel in 0.5× TBE buffer. Gel was run in 0.5× TBE for 15 min. Electrophoretic mobility of the siRNA-nanoparticles was analyzed on a gel imaging system (Azure 400, Azure biosystems).

### Stability of siRNAs in serum

The stability of the siRNAs in nanoparticles was confirmed using agarose gel electrophoresis. The nanoparticles in 90% (v/v) human serum were incubated at 37°C, and each sample was collected at 24, 48, 72 and 96 h. The siRNAs were dissociated from the nanoparticles by adding 2-fold concentration of heparin to siRNA and incubating at 37°C for 30 min. After mixing the loading dye, samples were loaded into 2% agarose gel with 0.5× TBE. The gel was run for 15 min at 100 V. The siRNAs dissociated from the nanoparticles in serum were analyzed on a gel imaging system.

### Syngeneic orthotopic HCC tumor model

The mouse HCC cell line Hepa1–6 was modified to constitutively express luciferase (Lux). Hepa1–6 lux cells were initially grown in a donor female mouse (C57BL/6J) until the tumor was large enough to dissect. At this time, the donor mouse was sacrificed, the tumor excised and minced and then administered to naïve mice. Under anesthesia mice were placed in a supine position. A small transverse incision below the xiphoid was made to expose the liver. 1 × 10^6^ Hepa1–6 Lux cells suspended in 20 μl of Growth Factor Reduced Matrigel™ was slowly injected in the upper left lobe of the liver using a 29-gauge needle. The injection site was then covered using an absorbable gelatin sponge (AGS) and the liver was carefully placed back into the abdominal cavity so as not to disturb the AGS. The abdomen and the skin were then closed using a suture line and clips. Peri- and post-operative analgesia was administered; further post-operative analgesia was administered as deemed necessary. By administering luciferin intravenously in these animals, the luminescent signal produced within the tumor cells [tumor associated bioluminescence (TABL)] acted as a marker of the amount of tumor present. Signal was measured using an in vivo imaging system (IVIS) comprising a digital camera and quantitation software to measure the light emitted, which correlated with the size of the tumor.

### Drug administration

Once the tumors reached a TABL of ∼1 × 10e6 p/s (as determined by the luminescent signal generated), animals were randomized based on the signal and assigned to treatment groups representing day 0 of treatment. At this time point, and at given intervals afterwards, the drugs were administered IV by tail vein injection. Tumor size was subsequently measured by monitoring the luminescence signal every 2–4 days after injection.

### Quantitation of CD4+ and CD8+ T-cells

At discrete times post-treatment, animals were sacrificed, and livers removed. The excised livers were sectioned and either stained with hematoxylin and eosin (H&E), to view the tumor margins, or with mAbs specific for CD4+ or CD8+ T-cells. Imaging allowed the identification of the tumor margin and the generation of a mask with 25 um deep segments around the perimeter of the tumor, either in, towards the tumor, or out, away from the tumor. Quantitation of number of T-cells within each segment was made and this number could then be plotted by each segment and for each treatment.

### Uptake of siRNA by cells in the liver

All animal experiment protocols were approved by the Institutional Animal Care and Use Committee (IACUC) at the National Taiwan University College of Medicine. To determine the distribution of the PNP delivered siRNAs within the liver, we utilized a fluorescently labeled siRNA (Cy3 dye) administered systemically to mice. Each animal was injected IV with 150 μl Cy3-siRNA/HKP(+H) solution (equivalent to 15 μg siRNA) through the tail-vein. This was performed by IV administration of siRNAs formulated with Histidine Lysine Polypeptide (HKP(+H)) into naïve animals followed by sacrificing the animals at given times post administration (0, 2, 4, 24, 48 h).

Mouse liver was digested with 40mL Ca^2+^/Mg^2+^-free HBSS and followed by 50 ml 0.05% collagenase solution (Sigma-Aldrich, C5138) to obtain a single cell suspension. Crude non-parenchymal cells (NPCs) were separated from hepatocytes by low-speed (50×g) centrifugation. Crude NPCs were further purified by 25%/70% Percoll gradient at 1350×g for 30 min to remove red blood cells and cell debris. The isolated NPCs were stained with fluorochrome-conjugated antibody to discriminate each cell subpopulations. The antibodies used in this study included APC-CD45 (30-F11), PerCp-Cy5.5-F4/80 (BM8), PE/Cy7-CD11b (M1/70), FITC-CD146 (ME-9F1) and were purchased from Biolegend (San Diego, CA). Flow cytometry analysis was conducted by BD FACS Fortessa analyzer analyzed using FlowJo V10 software. Details of the separation of different cell types from the liver and their quantitation are shown in supplementary figures.

### Statement of ethics

The PNP distribution study in mice was approved and conducted in the animal center at National Taiwan University College of Medicine. All mice received humane care according to the guidelines established by the Institutional Animal Care and Use Committee (IACUC# 20210108). The protocol was specifically applicable to the experiments. All syngeneic orthotopic animal studies were performed at Crown Bio (UK). The work was performed under UK Home Office license PD26EF7AA, protocol 9 (Intra-hepatic tumor models). The experiments were performed under the conditions specified in the protocol and are applicable to the experiments reported.

## Results

### SiRNAs against TGFβ and COX2 show inhibition of their targets

25mer siRNAs were designed against each gene looking for conservation of gene sequence across multiple species (human, monkey, mouse and pig) (Supplemental Materials—Figure S1a). Notably, TGFβ was a match for all animals but differed from the mRNA in pig by one base (96% match). Cox 2 siRNA matched all species 100%. These siRNAs were tested in a concentration-response to determine their effects on silencing their target genes in human liver cell lines (HepG2 and SNU182) and mouse Hepa1–6 cell line. Supplemental Materials—Figure S1b and c shows the results of the siRNAs on silencing their respective genes in these cells. They both showed target silencing in a concentration-dependent manner. TGFβ showed half-maximal inhibition at >0.028 nM in HepG2 cells and ∼0.032 nM in Hepa1–6 while COX2 showed half-maximal inhibition at >0.41 nM in SNU182 cells and ∼0.27 nM in Hepa1–6 cells.

To get uptake of siRNAs into specific cell types, we utilized a polypeptide nanoparticle delivery system comprising a branched copolymer of Histidine and Lysine peptides (H3K4b (or HKP)) or H3K(+H)4b (or HKP(+H)). The sequence of HKP(+H) is [KHHHKHHHK**H**HHHKHHHK)_4_**Lys**. The additional Histidine moiety in HKP(+H) compared to HKP is indicated in bold type. For the branched peptide, the Lys shown in bold represents the three-lysine core from which the terminal branches are attached for both peptides (11,13,15–18).

When siRNAs are rapidly mixed with this branched polypeptide, they spontaneously form nanoparticles with a diameter of between 50 and 65 nm and a zeta potential of 33–38 mV (Supplemental Materials—Figure S1d). Using a microfluidic mixer, and the optimal peptide to siRNA ratio, the optimal siRNA concentration, and the optimal flow rate, the nanoparticles have a low PDI (polydispersity index) indicating very uniform manufacture. This polypeptide has been used extensively to deliver siRNAs into a variety of tumors and tissues (12,14,15,19) where it demonstrates silencing. We evaluated the ability of the PNP containing siRNAs against TGFβ and Cox2 to silence the genes in a skin explant and the result was that PNP containing the two siRNAs dosed at 1mg/kg yielded about 50% silencing of their respective target genes at 48 h (Supplemental Materials—Figure S1e). Non silencing siRNA control (NC) had no effect on gene silencing (Supplemental Materials—Figure S1e).

We examined the distribution of fluorescent siRNA administered in PNP nanoparticles in mice and showed that the siRNA was found mainly in the liver, spleen and lung between 30 min and 6 h after administration IV (Supplemental Materials—Figure S1f). No siRNA was found in the heart or kidney at 6 h (Supplemental Materials—Figure S1f).

We saw silencing of the two genes (TGFβ and COX2) in the liver and lung of non-human primates administered the PNP formulated siRNAs at 2 mg/kg after a total of 9 administrations IV BIW (Supplemental Materials—Figure S1g).

### Uptake of siRNA nanoparticles into cells within the liver

**Figure 1.**
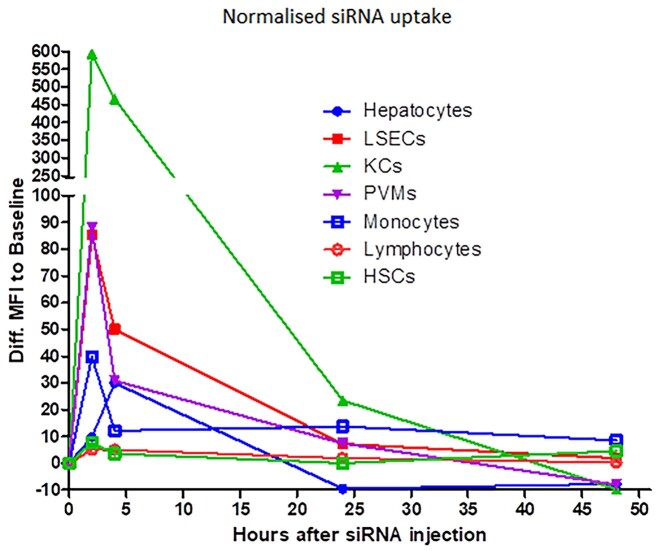
Distribution of IV administered siRNA formulated in PNP in cells in the liver. Fluorescent siRNA (Cy3-siRNA) in PNP nanoparticles was administered intravenously into naïve mice (15 ug per animal) which were then culled at the indicated times post administration. Livers were removed and the cells containing the labeled siRNA were quantitated after dissociation by using flow cytometry as detailed in the Methods. Graph shows time versus absorbed Cy3 fluorescent intensity in all hepatic cell subsets. The Y-axis indicates the difference between the Cy3 fluorescent intensity of baseline sample average and each sample taken at subsequent time-points. The value represents the quantity of net Cy3 absorbance of each sample. Four animals were included in each treatment group. Abbreviations used: LSEC, liver sinusoidal endothelial cells; KC, Kupffer cells; PVM, perivascular macrophage; HSC, hepatic stellate cells.

All mouse experiments were approved by the Institutional Animal Care and Use Committee (IACUC) of National Taiwan University. When delivered IV, the Polypeptide Nanoparticle (PNP) was previously shown to go to cells in the liver (Supplemental Materials—Figure S1f). We performed studies to determine the cells within the liver that take up the siRNA (described in Supplemental Materials). At 1h post transfection, the majority of the PNP, containing a fluorescent labeled siRNA, were located in Kupffer cells (KCs) and Liver Sinusoidal endothelial cells (LSECs) (Figure 1). The LSECs showed significant Cy3-siRNA uptake at 2–4 h after HKP(+H)-siRNA complex injection. Approximately 40% of LSECs exhibit Cy3-siRNA uptake at 2 h, and the frequency decreased to 20% at 4 h. The Cy3 fluorescence returns to baseline 24 h after drug injection. The result indicates that HKP(+H)-siRNA complex may target 20–40% of LSECs. The KCs showed significant Cy3-siRNA uptake at 2–4 h after HKP(+H)-siRNA complex injection. Similar to LSECs, the Cy3 fluorescence in KCs returns to baseline 24 h after drug injection. The result indicates the HKP (+H)-siRNA complex may target 50–55% of KCs. The Periventricular Macrophages (PVMs) showed significant Cy3-siRNA uptake at 2–4 h. The result indicates that HKP (+H)-siRNA complex may target 20–40% of PVMs. Further details of the discrimination of liver cells and quantitation of Cy3 siRNA uptake is available in the supplementary materials.

**Figure 2.**
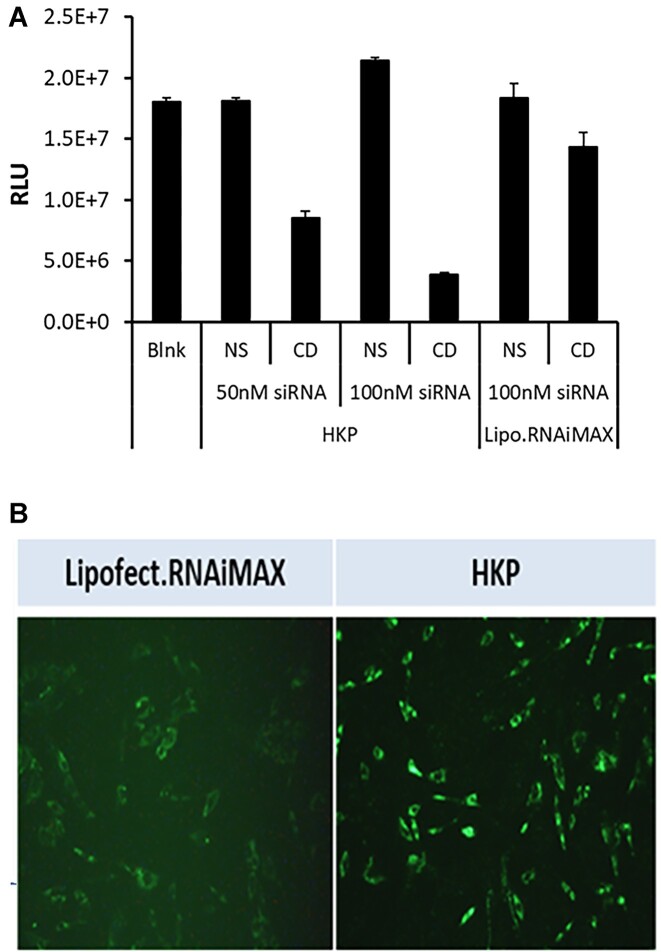
(**A**) Stellate cell transfection monitored by cell viability. To validate cellular uptake of siRNA by PNP we compared the efficacy of transfection driven by PNP or Lipofectamine RNAiMAX in human primary hepatic stellate cells in culture. Stellate cells were plated in 96 well plates at a density of 1.2 × 10^3^ cells/well and were allowed to settle for 24h. At this time, non-silencing siRNA (NS) or Cell Death SiRNA (CD; Qiagen, Germantown, MD) were added—either at 50 nM or 100 nM in PNP, or at 100 nM in lipofectamine RNAiMAX. A control (Blank) was not treated with any reagents. After a further 72 h incubation, cell viability was monitored using CellTiter-Glo(R)-2.0 reagent (Promega, Madison, WI) by measuring luminescence signal. The figure shows the luminescence signal plotted for each treatment. Data is plotted ±SEM (*n* = 4 points). (**B**) Uptake of fluorescent siRNA by stellate cells. Using lipofectamine or PNP, we examined uptake of AF647 fluorescently labeled siRNA into human hepatic stellate cells in culture. Cells were plated in 96-well plates and were transfected with 50 nM fluorescently labeled siRNA mixed with Lipofectamine RNAiMAX (left panel) or PNP (right panel). After 24 h cells were imaged using a Cytation 5 instrument (BioTek, Winooski, VT) set up for monitoring fluorescence.

In the cell sorting experiment, the hepatic stellate cells (HSC) represented a very low percentage of the overall cell population within the liver and the signal within these cells for the fluorescent labeled siRNA was too low to quantify additional changes. We therefore examined the effect of transfection on primary human hepatic stellate cells independently (Figure 2A). HKP mediated delivery of a fluorescent (AF647) labeled NS siRNA was compared with transfection by lipofectamine RNAiMax of the same siRNA in human primary hepatic stellate cells (Figure 2B). It was observed that HKP resulted in a greater fluorescent signal in the human hepatic stellate cells than the lipofectamine reagent (Figure 2A). Furthermore, we confirmed this result by using Non silencing siRNA (NS) and Cell Death SiRNA (CD (Qiagen, Germantown, MD)) transfected with the two reagents. HKP mediated a greater effect of the CD siRNA than Lipofectamine RNAiMax after 72h incubation (Figure 2A) demonstrating effective delivery of siRNAs to hepatic stellate cells using this reagent.

### Syngeneic orthotopic model for testing efficacy of PNP delivery of TGFβ /Cox2 siRNA

All syngeneic orthotopic animal studies were approved by Crown Bio (UK) animal ethics committee and performed at Crown Bio (UK). Since we had demonstrated uptake of IV administered PNPs into the liver—with good distribution through the key cells in the liver—and, since HCC is one of the worst responding cancers to the immune checkpoint inhibitors, we decided to examine the effect of siRNA administration ± an anti-PDL1 mAb to treat liver cancer. We elected to use a syngeneic orthotopic HCC model developed by Crown Bio (UK). All *in vivo* experiments and imaging to quantitate the amount of CD4+ and CD8+ T-cells in and around the tumor were performed at Crown Bio (UK).

Tests were performed looking at Control alone (PNP + Non-Silencing siRNA, IV BIW), Sorafenib (50 mg/kg, PO QD), anti-PDL1 alone (5mg/kg, IP BIW), anti-PDL1 (5 mg/kg, IP BIW) + PNP + TGFβ/Cox2 siRNA (1 mg/kg, IV BIW), anti-PDL1 (5 mg/kg, IP BIW) + PNP + TGFβ/Cox2 siRNA (2 mg/kg, IV BIW), and PNP + TGFβ /Cox2 siRNAs alone (2 mg/kg, IV BIW).

**Figure 3.**
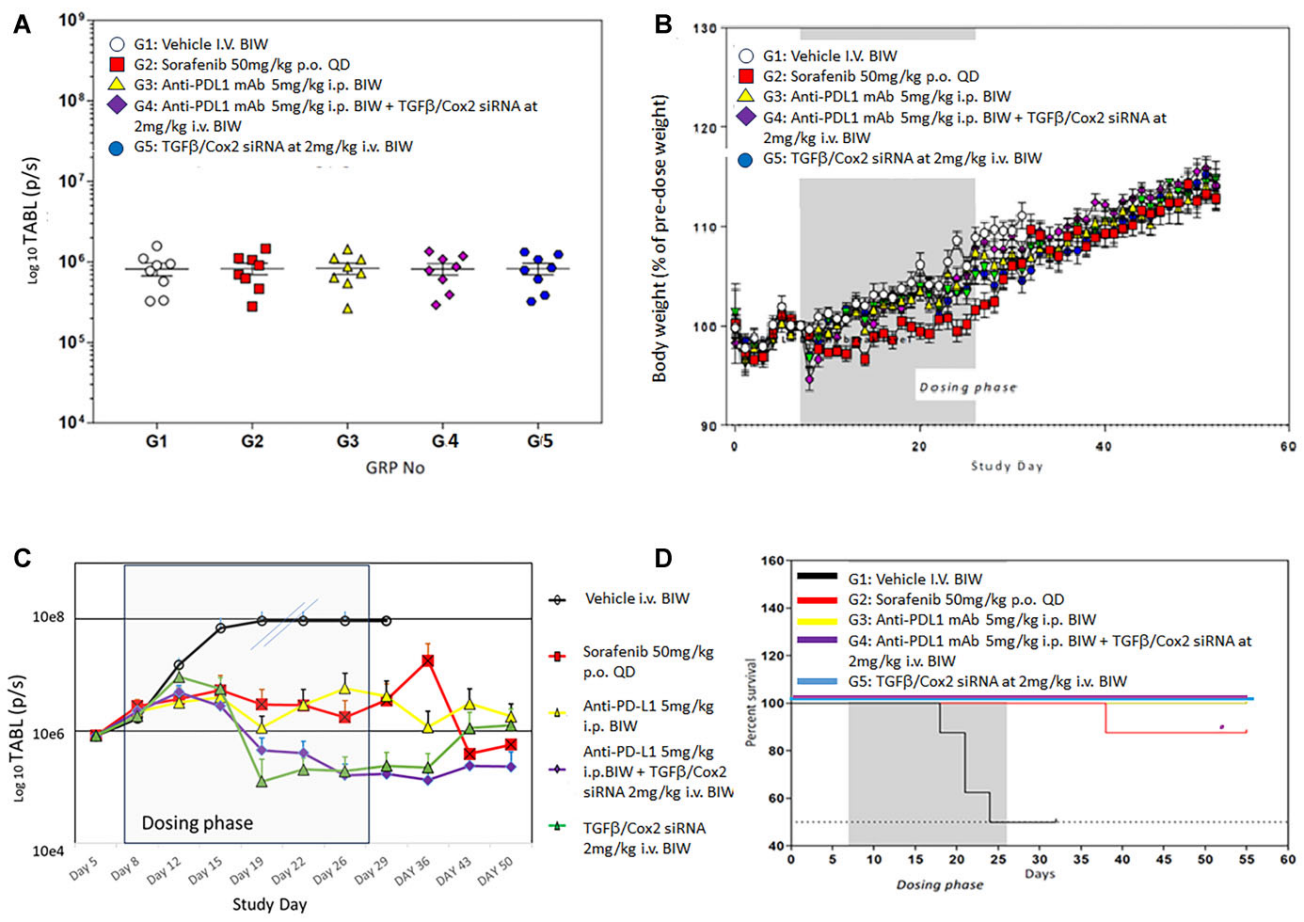
(**A**) Effect of TGFβ and Cox2 siRNA delivery in animals bearing tumors. Hepa1–6 cells expressing luciferase (Hepa1–6lux) were used in this syngeneic orthotopic model. Animals were treated as in Materials and methods. TABL (tumor associated bioluminescent) measurements of mice at study initiation. All animals were randomly allocated to the 6 different study groups (*n* = 8), according to TABL measurements. (**B**) Body weight measurements over the time-course of the study. Mice were weighed daily, and the weights recorded. The dosing phase of the study is indicated. Treatments were as follows: G1: vehicle alone, G2: sorafenib (50 mg/kg P.O. QD), G3: anti-PDL1 Ab (5 mg/kg I.P. BIW), G4: anti-PDL1 Ab (5 mg/kg I.P., BIW) + siRNAs against TGFβ and Cox2 (40 ug/injection (2 mg/kg IV BIW)) in PNP, G5: siRNAs against TGFβ and Cox2 40 ug/injection (2 mg/kg IV BIW). Only sorafenib caused an alteration in body weight but this did not reduce >10% over the time course of the study. Monitoring, after dosing ended, continued for an additional 30 days with no tumor recurrence observed. (**C**) Efficacy of treatments. All treatments were administered 6 times BIW over 3 weeks. Vehicle treatment (black, open circles) resulted in rapid growth of tumors. The double slashes are where the last observation carried forward (LOCF) was made due to the need to cull animals for ethical reasons. Sorafenib 50 mg/kg PO QD (red squares), anti-PDL1 mAb 5 mg/kg IP BIW (yellow triangles), anti-PDL1 Ab 5 mg/kg IP BIW + TGFβ/Cox2 siRNAs in HKP (2 mg/kg IV BIW) (purple diamonds), TGFβ /Cox2 siRNA in HKP (2 mg/kg IV BIW) (green triangles). The dosing phase of the experiment is indicated. Data is plotted ±SEM (*n* = 10 animals per data point). (**D**) Kaplan–Meier survival plot. The black line represents control (NS siRNA treated animals) where we see about a 50% reduction in mortality of animals over the dosing phase of the experiment (dark grey background). The red line represents Sorafenib treatment where we saw a single animal die after the conclusion of the dosing phase of the experiment. All other treatments (anti-PDL1, anti-PDL1 + TGFβ/Cox2 siRNA and TGFβ/Cox2 siRNA alone) resulted in no deaths (gold, purple and blue lines).

Animals were randomly assigned to the experimental treatment groups and the tumor associated bioluminescent intensity (TABL) emitted by the tumors was measured in each animal to ensure that the distribution was even. Figure 3A shows the even distribution after randomization across the treatment groups at study initiation.

As a measure of any adverse effects of drug administration on the animals, we monitored body weights throughout the time-course of the experiment. Figure 3B shows that only the Sorafenib arm showed any effect on body weights over the time-course of the experiment. However, none of the treated animals showed a loss >10% of body weight which would have resulted in a break from dosing and possible removal of the animal from the analysis.

As shown, in Figure 3C, we examined several different treatment regimens on the growth of the HCC tumors in the animals. The anti-PDL1 antibody was administered IP at 5 mg/kg while PNP nanoparticles containing TGFβ/Cox2 siRNA were administered at 2 mg/kg IV through the tail vein BIW. We used sorafenib (a tyrosine kinase inhibitor) as an appropriate control since this is the current small molecule standard of care for liver cancer.

TABL was measured at the days shown throughout the time-course of the experiment. Dosing was stopped at day 26. LOCF—last observation carried forward—was applied to the vehicle treated samples from day 19 since these tumors grew very large and animals were sacrificed for humane reasons.

The initial concept for this study was to evaluate the infiltration of T-cells into the tumors by conclusion of the dosing phase (26 days). However, as the data show (Figure 3C), we observed a reduction in tumor size, as evidenced by the reduction in TABL after treatment with PNP + TGFβ/Cox2 siRNAs alone, such that there were no tumors visible after the 6th dose (day 22). Consequently, we would not expect to see tumor, and therefore could not evaluate any improvement in T-cell penetration into the tumor. We therefore adapted the study to examine whether there was any sign of tumor recurrence after dosing had ceased. We monitored TABL signal from the animals to day 50 and tumor regrowth was significantly reduced in the positive siRNA treated animals. Notably, the addition of the immune checkpoint inhibitor, PD-L1 mAb, seemed to prolong the effect of TGFβ and COX2 siRNA on inhibiting tumor growth at days 43 and 50 (Figure 3C).

This effect translated into improvement in animal survival as demonstrated by the Kaplan Meier survival plot (Figure 3D). During the dosing phase, we saw 50% of the animals that were treated with a non-silencing (control) siRNA died by day 26 (Figure 3D). We lost one animal on the Sorafenib arm approximately 10 days after conclusion of dosing (Figure 3D). However, in all the other treatment arms animals remained viable and there was no mortality—even after cessation of treatment at day 26 and following the animals for a further 29 days (Figure 3D).

### Lowering the dose and number of administrations allowed recovery of tumor to measure T-cell penetration

To evaluate the effect of PNP nanoparticles + TGFβ/Cox2 siRNAs on T-cell infiltration into tumors, we altered the dosing regimen so that it was unlikely we would see a complete reduction of the tumors by the single agent siRNA administration.

**Figure 4.**
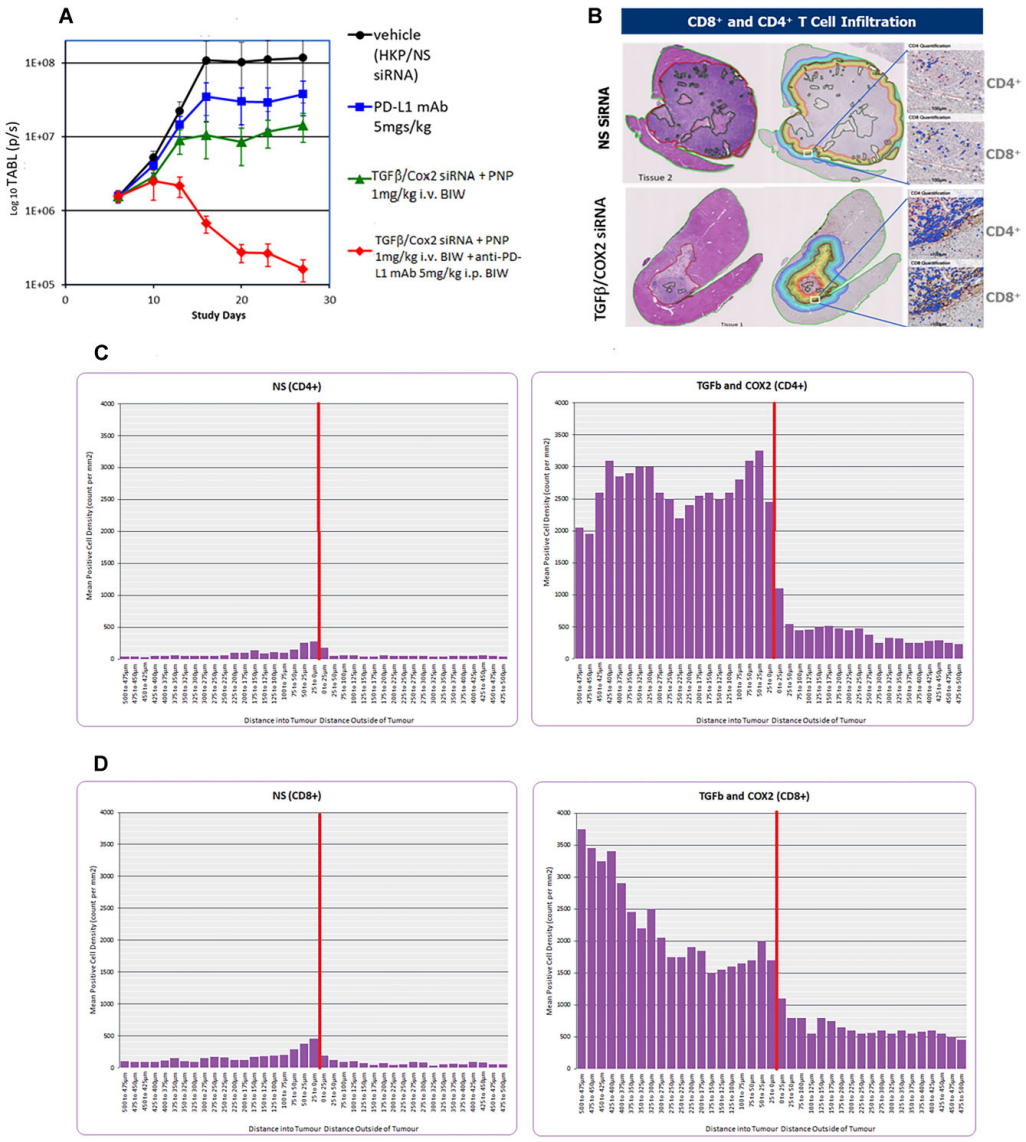
(**A**) Effect of lowering the dose of siRNA administered in PNP. Syngeneic orthotopic tumor growth was initiated as detailed in the Materials and methods. All treatments were administered six times BIW over 3 weeks. The animal exposure experiment was repeated at a lower dose of TGFβ/Cox2 siRNAs in PNP (1 mg/kg IV BIW). Vehicle (black circles) consisting of PNP + non silencing siRNA resulted in a significant growth of the tumor. PDL1 mAb alone (5 mg/kg IP BIW; blue squares). TGFβ /Cox2 siRNA in PNP (1 mg/kg IV BIW) (green triangles). TGFβ/Cox2 siRNA in PNP (1 mg/kg IV BIW) + anti-PDL1 mAb (5 mg/kg IP BIW) (red diamonds). Data is plotted ±SEM (*n* = 10 animals per data point). (**B**) Examples of recovered tumors from samples treated with non-silencing (NS) siRNA (upper panel) or TGFβ/Cox2 siRNA in PNP (lower panel). Animals were treated with NS siRNA or TGFβ/Cox2 siRNA in PNP at 1 mg/kg for just three doses (BIW) and then animals were sacrificed, and livers excised, fixed and sectioned, and sections either H&E stained or stained for CD4+/CD8+ T-cells. The left most sections are stained with H&E and show the dramatic reduction in tumor volume upon treatment with TGFβ/Cox2 siRNA + PNP. The rightmost sections show the regions identified where the tumor margin meets the liver. The different colored lines indicate segments around the tumor margin at 25 um intervals in towards the tumor or out away from the tumor. The white square in each figure shows a region examined for T-cell penetration as shown expanded in the right panels for CD4+ and CD8+ T-cells. (**C**, **D**) Staining for CD4+ and CD8+ T-cells in the liver sections. CD4+ (C) and CD8+ (D) were counted in the various segments and the values are plotted in the figures. The peritumoral zone is represented by the vertical red line and T-cell counts in each 25 um segment either, in (toward the tumor), or out (away from the tumor) were counted and plotted for non-Silencing siRNA (NS; left panel) or TGFβ and Cox2 siRNA (right panel). A representative image for each treatment was used to calculate the T-cell penetration but similar observations were made in other experiments.

To validate the response to TGFβ and Cox2 siRNA in PNP, we performed the experiment using the syngeneic orthotopic model but administration of the siRNA formulation at just 1 mg/kg BIW (not 2 mg/kg BIW as in Figure 3C). As with the previous model, PNP + non-silencing (NS) siRNA treatment alone resulted in a rapid growth of the tumor. PDL1 mAb (5 mg/kg IP BIW) had a small effect on the reduction of the rate of tumor growth. PNP nanoparticles + TGFβ and Cox2 siRNA alone at 1 mg/kg reduced the rate of growth of the tumor slightly more than the anti-PDL1 treatment alone after the administration of six doses (IV BIW). However, combining TGFβ and Cox2 siRNA and anti-PDL1 mAb resulted in a reduction in tumor size. This result confirmed the observed improvement of anti-PDL1 mAb by inclusion of TGFβ and Cox2 siRNA observed in the previous experiment (Figure 4A).

Therefore, in subsequent experiments aimed at detecting CD4+ and CD8+ T-cell penetration into the tumor, we treated the animals with just three doses of TGFβ and Cox2 siRNA (1 mg/kg IV BIW) and then sacrificed the mice, excised the livers, and sectioned and stained them.

We recovered the livers and observed that, while reduced, some tumor tissue remained (Figure 4B). The sections were stained with H&E to show the tumor location and size. TGFβ & Cox2 siRNA administration has an effect in reducing the size of the tumor. The margins of the tumor/liver interface are drawn in the right-hand image of the sections (Figure 4B) along with segments at 25 uM intervals, in towards the tumor, or away from the tumor and into the liver. The tumor is outlined by the dark red line. In separate sections, the samples were stained for CD4+ and CD8+ T-cells. The region highlighted by the white box (Figure 4B) shows the tumor margin and the inset images show staining and quantitation of CD4+ and CD8+ T-cells specifically in this region.

Using the stained samples, Crown Bio (UK) applied an image algorithm to detect CD4+ and CD8+ T-cells within 25 uM virtual segments around the tumor. Segments were made at 25 uM intervals around the tumor perimeter, from the dark red line outlining the tumor—either in towards the tumor, or out—towards the liver. Each segment was monitored for CD4+ and CD8+ T-cells and the counts expressed relative to the position of the tumor margin. Figure 4C shows the distribution of CD4+ T-cells in the NS control siRNA compared with the siRNA treated group (TGFβ and Cox2 siRNA). Figure 4D shows the distribution of CD8+ T-cells in livers from animals exposed to Non-Silencing (NS) siRNA or TGFβ and Cox2 siRNAs. CD4+ and CD8+ T-cells were increased both around the periphery of the tumor but also at greater depth into the tumor than in the NS treated samples.

Treatment with TGFβ and Cox2 siRNA in PNP resulted in an increase in both T-cell types at the tumor margin. CD4+ T-cells showed ∼3000 per sq. mm and were maintained at this high level for up to 500uM in towards the tumor (Figure 4C). CD8+ T-cells were also increased significantly at the tumor margin (∼2000 cells per mm2) relative to the non-silencing siRNA control and this number increased with depth into the tumor to ∼3500 CD8+ T-cells per mm2 at 500 uM away from the margin (Figure 4D).

When data from the treatments were compared based on distance from the interface, we saw an improvement in CD4+/CD8+ T-cell penetration into the tumor in TGFβ and Cox2 siRNA treated sample (Figure 4D).

## Discussion

We identified two 25mer siRNA sequences able to silence their respective targets (TGFβ and Cox2). TGFβ siRNA shows identity to human, mouse and monkey mRNA with just a single base mismatch to the pig. Cox2 siRNA is identical across all species. The sequences produce concentration and time-dependent reduction of their respective targets (Supplemental Materials—Figure S1b, c, e). When formulated into a polypeptide nanoparticle (PNP), the nanoparticles, containing an equimolar amount of each of the 2 siRNAs, are about 60 nM in size, and are uniform (measured by PolyDispersity Index or PDI; Supplemental Materials: Figure S1d1). The particles show a zeta potential of ∼35 mV (Supplemental Materials—Figure S1d1). Free siRNA is rapidly degraded in serum, but packaging in PNP, the nanoparticles protect the siRNAs from degradation by serum (Supplemental Materials—Figure S1d2). The additional Histidine in HKP(+H) compared with that in HKP has several benefits. It seems to release the siRNA more rapidly within the cell—possibly as a result of the protonation of the histidine moieties as the endosome acidifies (17,18) and it is possible that the additional histidine modifies the interaction between the peptide and the siRNA (by displacing the lysine interaction with the negatively charged phosphates along the backbone of the siRNA) and enhances more rapid release of siRNA from the nanoparticle (11,14,15). Furthermore, HKP(+H) shows low cytokine stimulation (lower than some liposomes (14,17).

Given IV, PNP formulations are rapidly taken up by the liver and we can show distribution into several key cells in the liver (Figure 1, Supplemental Materials—Figures S2–S10). Indeed, PNP delivery of siRNAs to the different cells in the liver, monitored by flow cytometry, showed rapid uptake by Kupffer cells, Liver Sinusoidal Endothelial cells and Perivascular Macrophage cells (Figure 1).

We could not determine uptake into HSCs since these represent only ∼1% of all cells and the signal was too low to provide accurate quantitation. However, we studied PNP transfection of specific human primary stellate cells and saw that siRNA was readily able to enter these cells with PNP. Furthermore, we showed that when we compared transfection of cell death (CD) siRNA on viability (Figure 2A), PNP delivered siRNA resulted in greater cell death than with Lipofectamine RNAiMax. We also showed that PNP produced a brighter fluorescence signal than Lipofectamine in these same cells when delivering fluorescent siRNA (Figure 2B).

While we demonstrate siRNAs formulated with HK peptides inhibit HCC, several studies have reported siRNA encapsulated in lipid-based nanoparticles to inhibit tumor growth in the liver. For example, Li *et al.* (20) examined a number of siRNA targets formulated in lipid nanoparticles and selected the best siRNA candidate targeting CDCA1 that resulted in a significant reduction in tumor growth in orthotopic models of HCC. Another study used siRNA targeting survivin in liposomes to inhibit HCC development (21).

TGF-β1 is the predominant isoform in the healthy liver and is mainly expressed by Kupffer cells and stellate cells. In fibrotic conditions, TGF-β1 levels increase significantly and are found to be expressed by most sinusoid cells (22). Furthermore, TGFβ plays a key role in tumor-stroma crosstalk (23–28) and activation of HSCs leads to secretion of cytokines (including TGFβ, stromal derived factor (SDF), platelet derived growth factor (PDGF), HGF and ECM proteins, fibronectin and collagen) (24,26–29). These agents induce tumor cell growth and provide a physical barrier for the immune system, leading to exclusion of CD8+ T- cells (30,31). In later stage tumors responsive to TGFβ, the cytokine can also induce Epithelial Mesenchymal Transition (EMT) (32), which has also been shown to contribute to chemo-resistance and immune evasion (33). TGFβ also affects CD8+ T cells directly by blocking T cell receptor-mediated T cell activation (34).

Induction of cytostatic and apoptotic pathways causes T cell numbers to be reduced. Using a nanoparticle formulation of siRNAs (targeting TGF-β and COX2) at 2 mg/kg IV BIW in a syngeneic orthotopic liver cancer model, we demonstrate rapid reduction of the tumor over the dosing period (six doses BIW; Figure 3C). We further showed that this treatment produced an antitumor response—resulting in no deaths for at least 30 days after dosing was stopped (Figure 3D). Silencing of TGFβ and Cox2 at this siRNA concentration reduced tumor, but at 43 and 50 days we see that inclusion of anti-PDL1 mAbs further reduces tumor viability compared to siRNAs alone (Figure 3C). While PNP + TGFβ/COX2 siRNA (1 mg/kg i.v. BIW) had a significant effect on its own, it was not sufficient to inhibit the tumor completely (Figure 4A). Consequently, when combined with anti-PDL1, we saw a more pronounced effect, further suggesting that the addition of the immune checkpoint inhibitor augmented the effect on tumor reduction. In subsequent experiments, we selected the 1 mg/kg dose of PNP and siRNA and reduced administration to just 3 times (BIW) to still have tumor at the conclusion of treatment. Using this regimen, we were able to see tumor growth inhibition while retaining a large enough tumor to be able to quantitate T-cell penetration (Figure 4B). This allowed us to determine that the effect was correlated with an increase in T-cell penetration into the tumor (Figure 4C, D) as demonstrated for colorectal and urothelial metastatic cancers in the liver when inhibiting TGFβ using the small molecule, galunisertib (6,10).

By Including a TGFβ siRNA and a Cox 2 siRNA in a single nanoparticle for systemic delivery and monitoring its effect on HCC in a mouse model, we benefit from silencing each target gene which enables greater penetration and intra-tumoral trafficking of CD4+ and CD8+ T cells.

Previous observations have indicated that CD8+ cells were found to occur in HCC, with highest numbers at the invasive margin (35).

TGFβ has been shown to play a significant role in the etiology of cancers (2–4,7,35–44). TGFβ is often overexpressed in cancers including HCC (1,35,38), and HCC may promote TGFβ production by the tumor microenvironment (TME) (2–4). Higher levels of TGFβ signaling in the Tumor MicroEnvironment (TME) is associated with poor prognosis (3,4,45). TGFβ may also be produced by liver sinusoidal endothelial cells and Kupffer cells (24–26), also targeted by the PNP – delivered siRNA.

TGFβ signaling in the tumor microenvironment is a determinant of tumor T cell exclusion and a consequent poor response to PD-1/PD-L1 inhibition (6,10).

Blocking TGFβ signaling in CAFs and T-cells, using galunisertib, significantly reduced metastasis formation by enhancing T- cell-mediated tumor cell killing in early metastatic stages (10).

Consequently, reducing TGFβ signaling, has been demonstrated to promote anti-tumor immunity as a monotherapy and to produce durable, complete responses, in combination with checkpoint blockade (5). Thomas and Massagué showed that systemic neutralization of TGFβ in vivo results in tumor eradication, associated with an increase in CD8+ T-cell mediated tumor-cell-specific cytotoxicity (44). Inhibition of TGFβ may also have antitumor effects in HCC (1,5,42–44).

We see single agent action of PNP nanoparticle delivered siRNAs targeting both TGFβ and Cox2 – not only inhibiting orthotopic HCC tumor growth but producing tumor regression. This suggests that silencing TGFβ and Cox2 exhibits a therapeutic effect.

COX-2 expression is very low in normal liver tissues but is elevated in HCC tumors (46,47). Cox2 produces prostaglandin E2 (PGE2), which suppresses immunity and fuels tumor-promoting inflammation (8,9). Cyclooxygenase (COX)-1 and 2 (the enzymes critical for production of PGE2), are overexpressed in several tumor types (47,48). Inhibition of Cox2 (using Celecoxib) in HCC cell lines was shown to inhibit their growth (47,49). Cox2 inhibition has also previously been shown to augment immunotherapies (8,9). In this regard, Zelenay *et al.* (8), showed that PTGS2 mRNA levels were inversely correlated with CD8 transcript levels, a measure of the presence of CD8+ T cells in tumors that was associated with longer survival and favorable treatment outcome (50,51).

Furthermore, inhibition of COX synergizes with anti-PD-1 blockade in inducing eradication of tumors, implying that COX inhibitors could be useful adjuvants for immune-based therapies in cancer patients (8).

PD-L1 is a ligand for PD-1 that is expressed on activated CD8+ T cells and some other immune cells, such as macrophages. PD-L1 binding to PD-1 inhibits cytotoxic T cell activity and promotes other immuno-inhibitory effects (52). PD-L1 expression by neoplastic cells and inflammatory cells in the tumor microenvironment (TME) of HCC is significantly correlated with markers of tumor aggressiveness (53), and increased PD-L1 expression in HCC is associated with poor prognosis (54,55) and disease recurrence (56). PD-L1 is detectable by IHC in non-tumor liver tissue and is mainly located in immune cells (e.g. Kupffer cells), in endothelial cells of central veins, and microvessels in the liver tissue, as well as in the intratumoral microvasculature (35).

PD-L1 is implicated in immune suppression in HCC by its presence in tumors and adjacent tissue, and high PD-L1 expression in HCC has been positively correlated with poor Barcelona Clinical Liver Cancer scores, portal vein invasion, and reduced overall survival (57).

By reducing the dose and number of administrations of TGFβ and Cox2 siRNA (3 doses), we could demonstrate the reduction in the tumor was concomitant with an increase in infiltration of CD4+ and CD8+ T-cells into the tumor (Figure 4B–D). We further demonstrated that the CD8+ T-cells were increased at greater depth into the tumor when the siRNAs were administered (Figure 4D).

These observations are important since immune checkpoint blockade with antibodies results in tumor stabilization or shrinkage in only ∼15–40% of patients (36,53,58). Therefore inhibition of TGFβ and COX2 by siRNA may represent a new therapeutic to improve the outcomes for patients treated with these agents.

Ganesh and Massague (36), identified that many tumors display an ‘immune excluded’ phenotype, in which T cells are limited to a peritumoral zone rich in fibroblasts and are reduced within the tumor itself. By inhibiting the expression of TGFβ and Cox2 within the liver we may convert immunologically cold tumors to ‘hot’ tumors where effector T-cells can penetrate deeper into the tumor and improve the efficacy of immune checkpoint inhibitors (1,36). This may produce a ‘T-cell inflamed’ phenotype with the deeper penetration of CD8+ T-cells into the tumor.

We are continuing the use of PNP nanoparticle delivered TGFβ and Cox2 siRNAs in a clinical trial in patients with liver metastases from the colon, pancreas, and other organs as well as in primary liver cancer. The Phase 1 clinical trial—a multi-center, open label, dose escalation and dose expansion study—is evaluating the safety, tolerability, and anti-tumor activity of PNP delivered siRNA (NCT 05037149). The current work also supports co-administration of an immune checkpoint inhibitor, such as PDL1 mAb, to improve efficacy and this will be the focus of future clinical trials.

## Supplementary Material

zcad059_Supplemental_File

## Data Availability

All data generated or analyzed during this study are included in this article and its supplementary material files. Further enquiries can be directed to the corresponding author.
