# Supplementary material for: Codelivery of TGFβ and Cox2 siRNA inhibits HCC by promoting T-cell penetration into the tumor and improves response to Immune Checkpoint Inhibitors
Source: NAR Cancer. 2024 Jan 9;6(1):zcad059. doi: 10.1093/narcan/zcad059 (PMC10776204; doi:10.1093/narcan/zcad059)
Supplement: zcad059_Supplemental_File [file zcad059_supplemental_file.docx]

**Supplementary Material; Uptake of nanoparticle packaged siRNA by the constituent cells in the Liver of C57BL/6J Naive Mice**

**For manuscript:**

**Codelivery of TGFβ and Cox2 siRNA inhibits HCC by promoting T-cell penetration into the tumor and improves response to Immune Checkpoint Inhibitors.**

Wookhyun Kim^1^, Zhou Ye^1^, Vera Simonenko^1^, Ashirwad Shahi^1^, Asra Malikzay^1^, John J. Xu^2^, Alan Lu^1^, Jau-hau Horng^3^, Chang-Ru Wu,^3^ Pei-Jer Chen^3^, Patrick Y. Lu^1^, David M. Evans^1^

1. Sirnaomics Inc., 20511 Seneca Meadows Parkway, Suite 200, Germantown, MD, USA 20876
2. Suzhou Sirnaomics Pharmaceuticals, Ltd., Biobay, Suzhou, China
3. National Taiwan University College of Medicine, No. 1, Section 1, Ren'ai Rd, Zhongzheng District, Taipei City, Taiwan 100

**Supplementary figures**

Fig. S1A. Alignment of siRNAs with their target gene in various species.

TGFβ is identical to mRNA for the gene in human, mouse and monkey but differs from pig at 1 base (shown by normal font).

Cox2 is identical to mRNA for the gene in all 4 species. All siRNAs used were chemically unmodified in their backbone. Non-silencing siRNA was used in control treatment in animal experiments.

Supplementary Fig S1a

|  |  |  |
| --- | --- | --- |
|  | Non-Silencing |  |
| SiRNA | **5'-CGAGCAGGGUAUCGACGAUUACAAA-3'** |  |
|  | TGFb1 | Cox2 |
| SiRNA | **5'-CCCAAGGGCUACCAUGCCAACUUCU-3'** | **5'-GGUCUGGUGCCUGGUCUGAUGAUGU-3'** |
| Mouse | **5'-CCCAAGGGCUACCAUGCCAACUUCU-3'** | **5'-GGUCUGGUGCCUGGUCUGAUGAUGU-3'** |
| Monkey | **5'-CCCAAGGGCUACCAUGCCAACUUCU-3'** | **5'-GGUCUGGUGCCUGGUCUGAUGAUGU-3'** |
| Human | **5'-CCCAAGGGCUACCAUGCCAACUUCU-3'** | **5'-GGUCUGGUGCCUGGUCUGAUGAUGU-3'** |
| Pig | **5'-CCCAAGGGCUACCAUGCCAA**U**UUCU-3'** | **5'-GGUCUGGUGCCUGGUCUGAUGAUGU-3'** |
|  |  |  |
|  |  |  |

Fig. S1.B.

TGFβ siRNA shows dose-dependent silencing of its gene. Expression was monitored in HepG2 cells and mouse Hepa1-6 cells in 12 well plates. SiRNA was transfected into cells using Lipofectamine RNAiMAX (Life Technologies, Carlsbad, CA) according to the manufacturer’s recommendations. 24h after transfection, total RNA was extracted using RNeasy Plus mini kit (QIAGEN), and expression monitored using qPCR as detailed in the methods. Data is plotted +/-SEM (n=3 points).

HepG2 cells


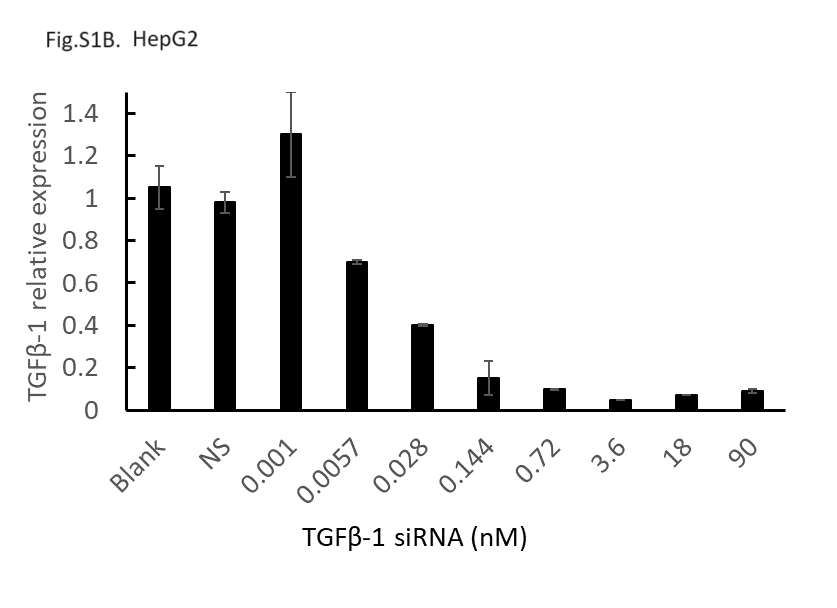


Hepa 1-6 cells


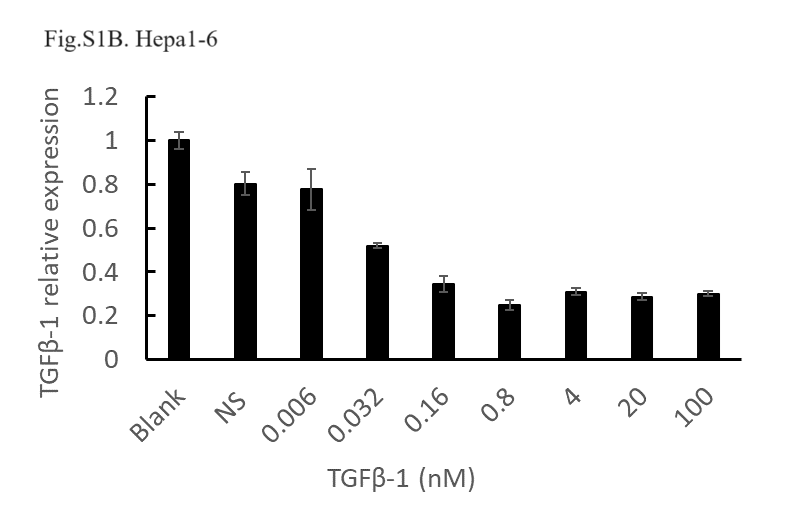


Fig. S1.C.

Cox2 siRNA shows dose dependent silencing of its gene. Expression was monitored in SNU182 cells and in mouse Hepa1-6 cells using the same method as for TGFβ. Data is plotted +/-SEM (n=3 points).

SNU182 cells


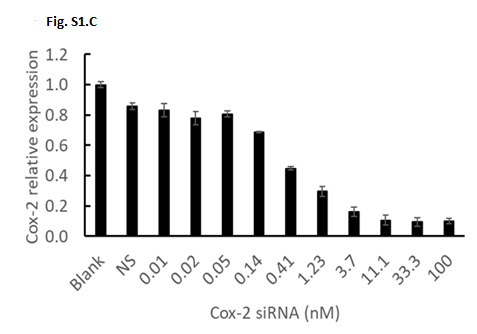


Hepa 1-6 cells


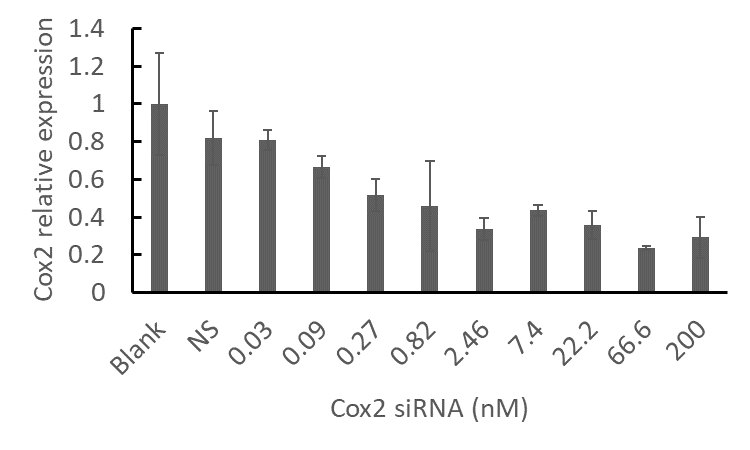


Fig. S1.D1.

Characterization of PNP formulations with siRNAs. The siRNA was mixed with each HK peptide (HKP and HKP(+H)) at a ratio of 2.5:1, 3:1, 3.5:1, and 4:1 (w/w) using a microfluidic mixer at 10ml/min. After incubation for 30 min, the size, PDI (PolyDispersity Index) and zeta potentials of the PNP formulations were measured using dynamic light scattering. Nanoparticle formulation was checked using a gel retardation assay for siRNA formulated with HKP (left) or HKP(+H) (right). Nanoparticles mixed with a 6 X loading dye were loaded into a 2 % agarose gel followed by electrophoresis at 100 V for 15 min, and the agarose gel was visualized through a gel imaging system. Data is plotted +/-SD (n=3 points).


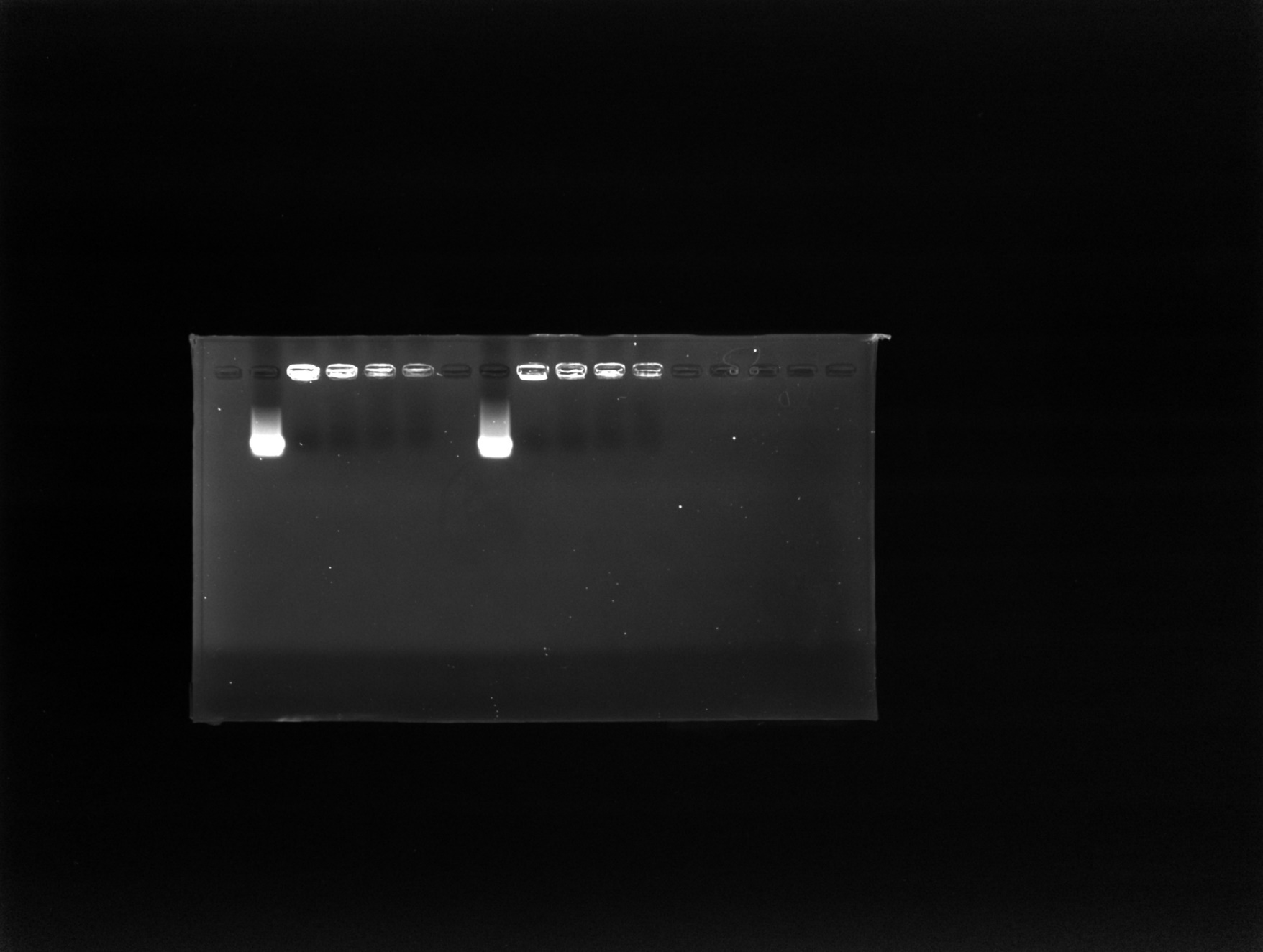


**2.5:1**

**3:1**

**3.5:1**

**4:1**

**Free siRNA**

**HKP/siRNA ratio**


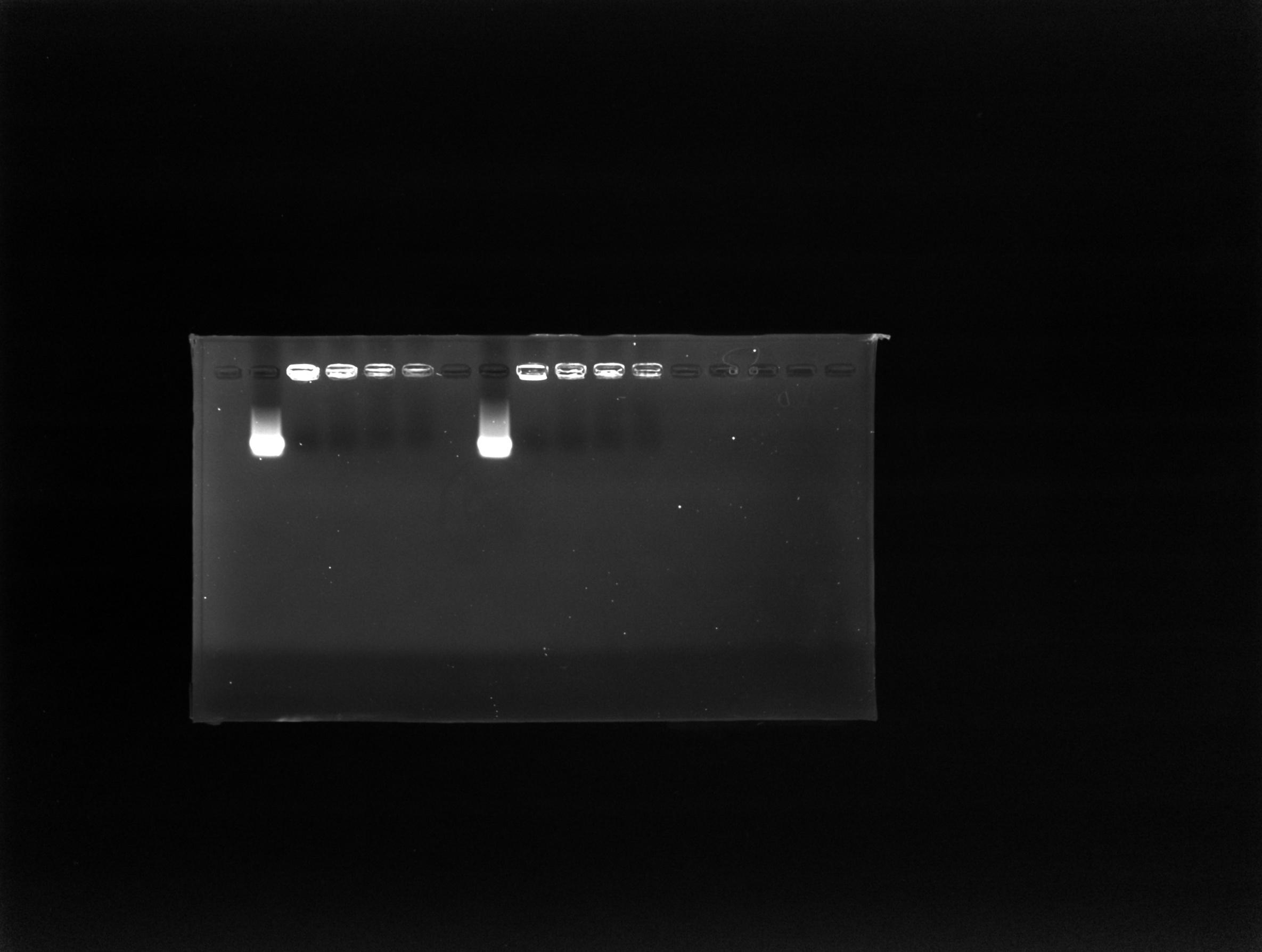


**HKP(+H)/siRNA ratio**

**2.5:1**

**3:1**

**3.5:1**

**4:1**

**Free siRNA**


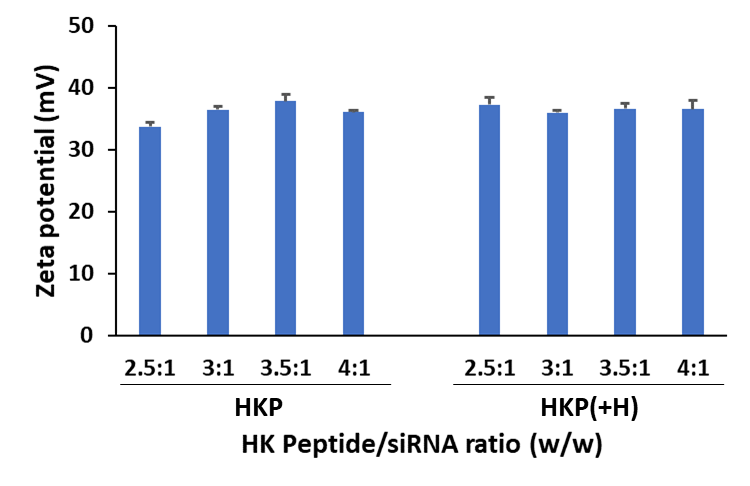

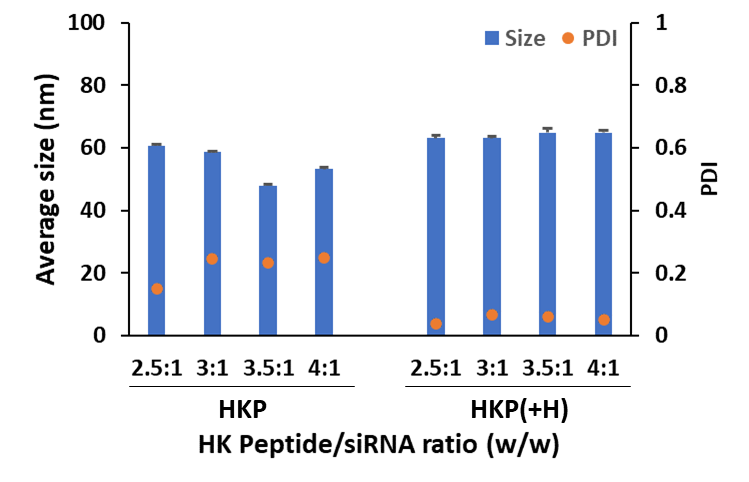


Fig. S1 D1.

Fig. S1D2.

siRNA stability was tested in serum. Gel retardation assay for stability of siRNA alone (left) and siRNA formulated with HKP (middle) or HKP(+H) (right) following treatment with human serum for 0, 4, 24, 48, 72, 96h (top images). Then heparin was added to dissociate the siRNA (bottom images). The mixture was loaded into a 2 % agarose gel followed by electrophoresis at 100 V for 15 min, and the siRNA band was visualized through a gel imaging system.


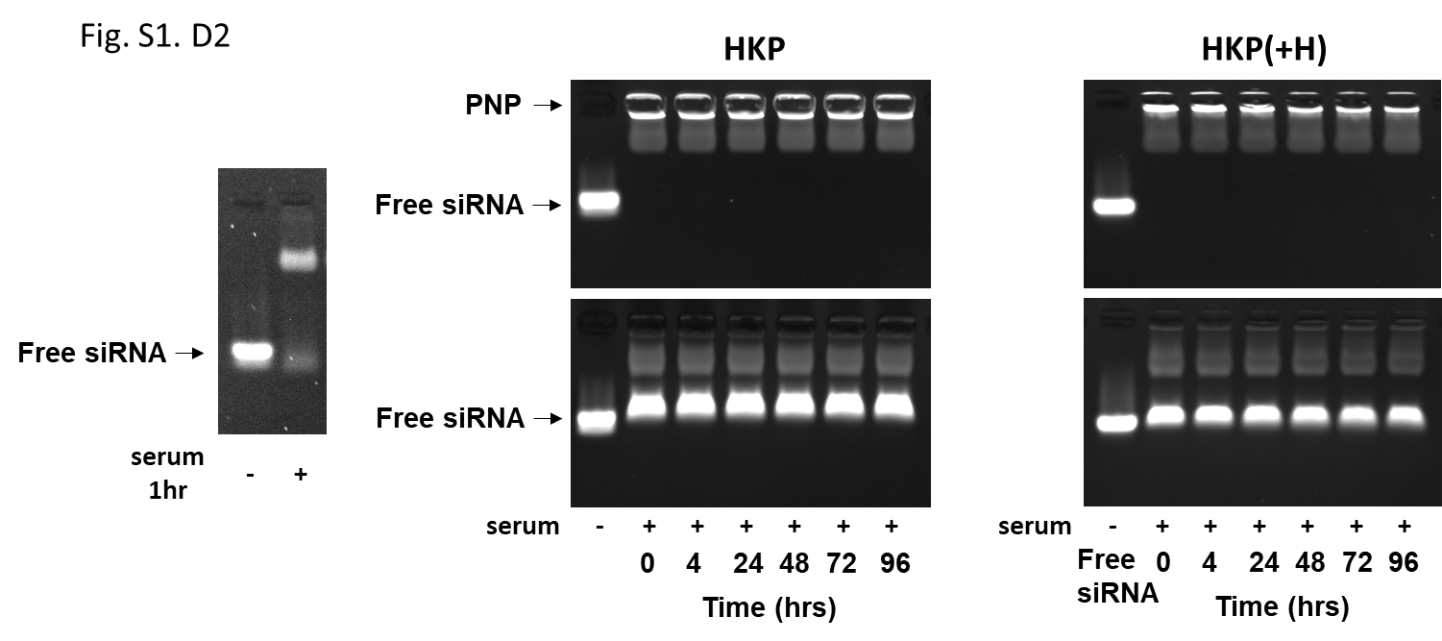


Fig. S1E.

Administration of 2 siRNAs results in silencing of their target genes. Silencing of TGFβ and Cox2 gene by siRNA administration to Human hypertrophic scar tissue implant in nude BALB/c mice using PNP. Human hypertrophic scar tissue implant in nude BALB/c mice were intra-scar injected with 2 or 4 μg of HKP + TGFβ/Cox2 siRNA. Sample was monitored for gene expression at 24h and 48h post-administration. TGFβ expression is shown (left panel) and Cox2 expression is shown (right panel) at 24h and 48h (Data is plotted +/- SD, n=4, *p=0.01).


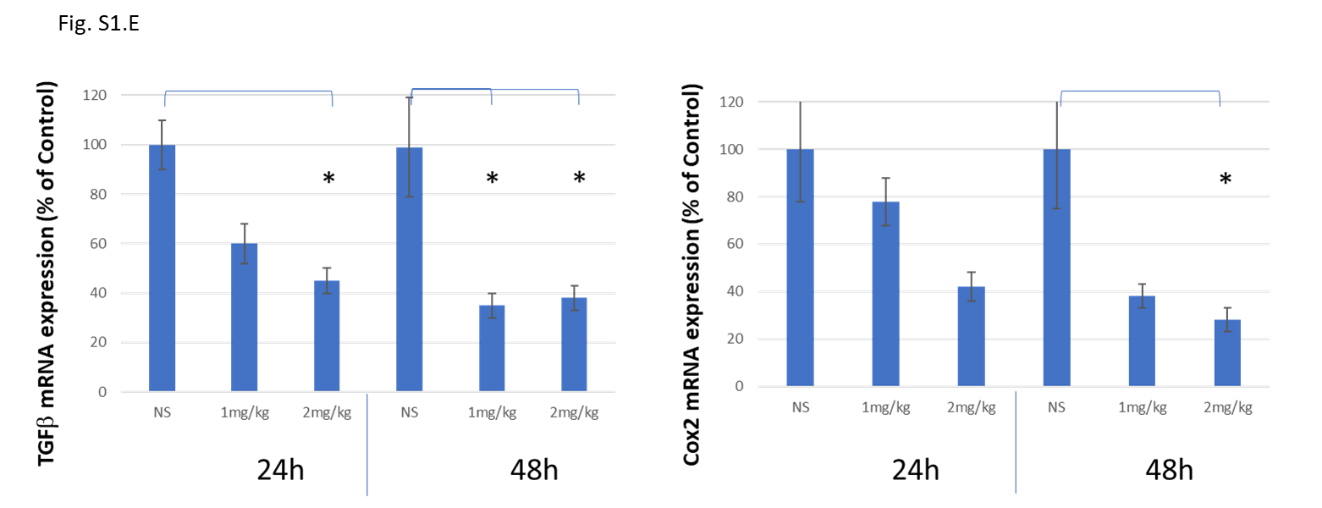


Fig S1.F. Distribution of fluorescently labeled siRNAs delivered using HKP polypeptide nanoparticles (PNPs) at 30mins-6hours after IV administration in a mouse.


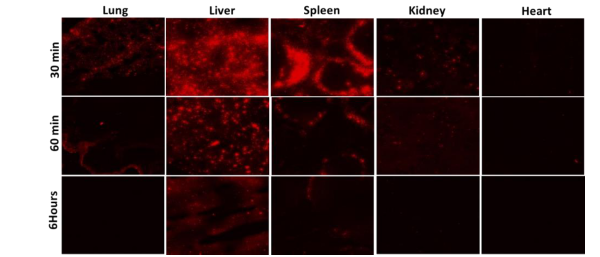


2mg/kg of PNPs (containing siRNA labeled with AF647 dye) were administered by IV (tail vein) injection. Animals were sacrificed at the time intervals shown and the various organs rapidly excised and used to evaluate the amount of fluorescently labeled siRNA that was present. SiRNA is rapidly taken up by the liver, spleen and lung. No siRNA was taken up by the kidney or the heart within 6h.

Fig. S1.G. SiRNA activity in NHP

TGFβ and Cox2 siRNA were administered IV in HKP(+H) in NHP at 2mg/kg 2x per week (4mg/kg/week) for a total of 9 times (n=4). On the day of the last dose (d30) tissues were collected and processed for qPCR. This data is the mRNA expression results of the target genes in liver and lung. Data is shown as the mean +/-sem for 4 animals.


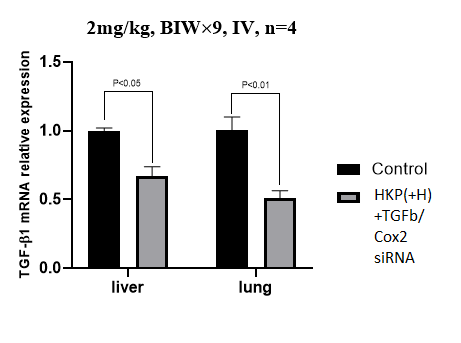


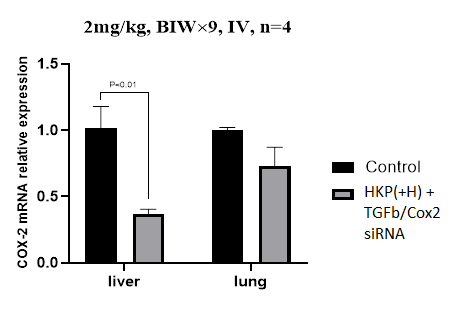


**Liver distribution of polypeptide nanoparticle formulated with siRNA.**

**1. Specific Aim and experimental design**

To understand the distribution and kinetics of siRNA-HKP(+H) nanoparticle in normal mouse liver, siRNA was conjugated with a fluorescent dye (Cy3) and packaged with HKP(+H). Two-doses of packaged Cy3-siRNA were administered to mice via intravenous route at 24 hours before cell isolation at 0 hour [Supplemental Materials Figure S2A]. Hepatic cells including the hepatocytes and non-parenchymal cells (NPCs) were isolated after livers were removed and the cells containing the labeled siRNA were quantitated following dissociation using collagenase digestion and Percoll purification at 2, 4, 24, and 48 hours post final siRNA injection [Supplemental Materials Figure S2A]. The cell isolation strategy is shown [Supplemental Materials Figure S2B]. Isolated hepatic cells were divided into 7 different subsets by specific surface markers by flow cytometry and the gating strategy is shown [Supplemental Materials Figure S2C and Table 1] (3) (4) (5). The uptake of siRNA within each cell type was measured by Cy3 fluorescence at each indicated time point. The cut-off value of Cy3 positive cells was set where the value of baseline samples has approximately 5% false positive cells.

**2. Materials and methods**

**2.1 Animals:**

Wild type C57BL6/J, female, 6 weeks-old were purchased from National Laboratory Animal Center (Taipei, Taiwan). Mice were housed in groups of less than 5 per cage in a standard specific-pathogen-free (SPF) grade facility. All animal experimental protocols were approved by the Institutional Animal Care and Use Committee (IACUC) at the National Taiwan University College of Medicine.

**2.2 Preparation and administration of Cy3-siRNA/HKP(+H) complex**

Silencer™ Cy™3-labeled Negative Control siRNA was purchased from Thermo Fisher Scientific. The Cy3-siRNA/HKP(+H) complex was produced by Sirnaomics (USA) and lyophilized in glass vials. Each vial contained 125μg Cy3-siRNA and was reconstituted with 625μL ddH_2_O to obtain 0.2μg/μL stock Cy3-siRNA/HKP(+H) solution. Stock solution was 1:1 diluted with 5% glucose solution before injection. Each animal was injected with 150μL Cy3-siRNA/HKP(+H) solution (equivalent to 15μg siRNA) through the tail-vein.

**2.3 Isolation of component cells: hepatocytes and liver non-parenchymal cells**

The cell isolation protocol is modified from a previously published protocol from Dr. Crispe (6). In brief, mouse liver was in situ digested with 40mL Ca^2+^/Mg^2+^-free HBSS and followed by 50mL 0.05% collagenase (Sigma-Aldrich, C5138) solution to obtain a single cell suspension. Crude NPCs were separated from hepatocytes by low-speed (50xg) centrifugation. Crude NPCs were further purified by 25%/70% Percoll gradient at 1350xg, 30 minutes to remove red blood cells and cell debris. The antibodies used in this study included APC-CD45 (30-F11), PerCp-Cy5.5-F4/80 (BM8), PE/Cy7-CD11b (M1/70), FITC-CD146 (ME-9F1) and were purchased from Biolegend and Flow cytometry analysis was conducted by BD FACS Fortessa analyzer and FlowJo V10 software.

**3. Results and analysis**

**3.1 Quality control of cell isolation procedure**

Before analyzing fluorescence inside the cells, we evaluated the quality of liver cell isolation by counting the isolated cell number. Among all tested groups, the average isolated hepatocyte count is approximately 80~100 million cells per mouse **[**Supplemental Materials - **Table 2]**, which is in accordance with previously published studies (7). Our isolated NPC number is approximately 8~10 million cells per mouse **[**Supplemental Materials - **Table 2]**, which fits a previously reported 10:1 hepatocyte to NPC ratio (8). No significant difference of isolated cell count was found among different time points. The result demonstrates good quality of cell isolation. The average analyzed cell number on flow cytometry is shown in Supplemental Materials - Table 3.

**3.2 Cy3-siRNA distribution in Hepatocytes**

The hepatocytes did not show significant Cy3-siRNA uptake after HKP (+H)-siRNA complex injection [Supplemental Materials Figure S3A-S3B]. However, a very minor hepatocyte population might uptake low-level siRNA 2-5 hours after injection. The result indicates the HKP (+H)-siRNA complex may not target hepatocytes.

**3.3 Cy3-siRNA distribution in Liver sinusoidal endothelial cells (LSECs)**

The LSECs showed significant Cy3-siRNA uptake at 2-4 hours after HKP (+H)-siRNA complex injection. Approximately 40% of LSECs exhibit Cy3-siRNA uptake at 2 hours, and this decreased to 20% at 4 hours. The Cy3 fluorescence returns to baseline 24 hours after drug injection. The result indicates that HKP (+H)-siRNA complex may target 20~40% of LSECs.

**3.4 Cy3-siRNA distribution in Kupffer cells (KCs)**

The KCs show significant Cy3-siRNA uptake at 2-4 hours after HKP (+H)-siRNA complex injection. Approximately 50~55% of KCs exhibit Cy3-siRNA uptake at 2-4 hours. Similar to LSECs, the Cy3 fluorescence in KCs returns to baseline 24 hours after drug injection. The result indicates that HKP (+H)-siRNA complex may target 50~55% of KCs.

**3.5 Cy3-siRNA distribution in perivascular macrophages (PVMs)**

The PVMs show significant Cy3-siRNA uptake at 2-4 hours after HKP (+H)-siRNA complex injection. The Cy3-siRNA dynamic in PVMs is similar to LSECs. Approximately 40% of PVMs exhibit Cy3-siRNA uptake at 2 hours, and this decreased to 20% at 4 hours. The Cy3 fluorescence returns to baseline after 24 hours. The result indicates that HKP (+H)-siRNA complex may target 20~40% of PVMs.

**3.6 Cy3-siRNA distribution in monocytes/PMNs**

The monocytes/PMNs showed modest Cy3-siRNA uptake at 2-24 hours after HKP (+H)-siRNA complex injection. Approximately 25% of monocytes/PMNs exhibit Cy3-siRNA uptake at 2 hours, and this decreased to 10% at 4 to 24 hours. The Cy3 fluorescence returns to baseline after 48 hours. This result indicates that HKP(+H)-siRNA complex may target 25% of monocytes/PMNs.

**3.7 Cy3-siRNA distribution in lymphoid cells**

The lymphoid cells showed minimum Cy3-siRNA uptake at 2 hours after HKP (+H)-siRNA complex injection. Only 8~10% of lymphoid cells exhibit Cy3-siRNA uptake at 2 hours, and this decreased to baseline after 4 hours. The result indicates the lymphoid cells are a poor target for HKP (+H)-siRNA delivery.

**3.8 Cy3-siRNA distribution in Hepatic Stellate Cells (HSCs)**

The HSC showed minimum Cy3-siRNA uptake at 2 hours after HKP (+H)-siRNA complex injection. Only 9~14% of HSCs exhibits Cy3-siRNA uptake at 2 hours, and this decreased to baseline after 4 hours. The result indicates that HSCs may be a poor target for HKP (+H)-siRNA delivery but experiments using primary human HSCs suggested otherwise (see manuscript Fig 2A and 2B).

**3.9 Comparisons of Cy3-siRNA uptake in different hepatic cell subpopulations**

To compare the dynamics of Cy3-siRNA uptake in cells after tail-vein injection, we plotted the time versus Cy3-positive cell frequency of all hepatic cells in a line-chart [Supplemental Materials Figure 10A]. The comparative result shows at 4 hours after drug injection, the HKP(+H) packaged siRNA can target more than 50% of Kupffer cells, and 15-20% of LSECs and PVMs. Only about 10% of monocytes/PMNs can take up Cy3-siRNA. The other cell types, including hepatocytes, lymphoid cells, and HSCs, have only minimal Cy3-siRNA incorporation. Cy3 fluorescent signal in all cell types returned to baseline 24 hours after injection, suggesting that the Cy3-siRNA in the cells degraded within 24 hours.

In addition to Cy3-positive cell frequency, we also analyzed the quantity of Cy3-siRNA uptake in each cell subpopulation. We calculated net Cy3-siRNA mean fluorescent intensity (MFI) by subtracting the average MFI of baseline group from each Cy3-siRNA injected samples. The time versus Cy3-MFI plot shows the quantity of Cy3 uptake in KCs outclasses all other hepatic cell types [Supplemental Materials Figure S10B]. The siRNA absorbance in LSECs and PVMs is weaker than KCs, but they still demonstrate clear siRNA uptake. The siRNA quantity in monocytes/PMNs is low, however, an obvious subpopulation shows clear fluorescent signal [Supplemental Materials Figure S7A], suggesting that this subpopulation could be targeted by the HKP(+H)-siRNA complex. The results are summarized in Supplemental Materials Table 4.

**Fig. S2. A**


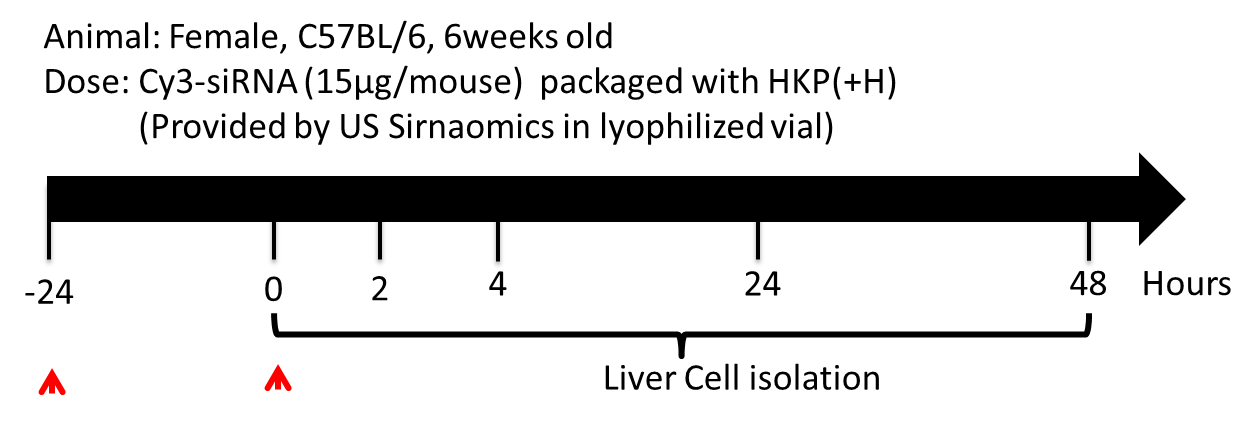


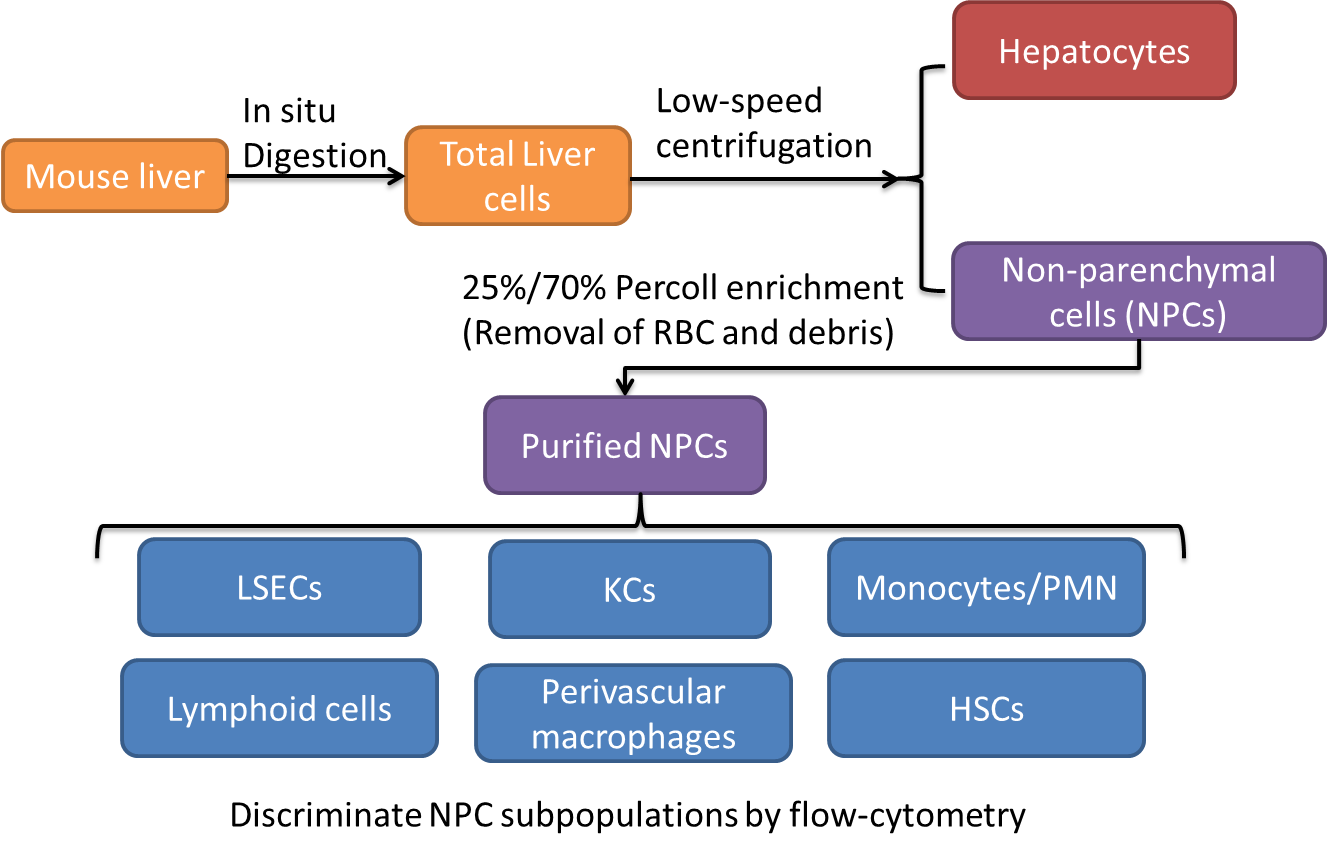


**Fig. S2. B**

**
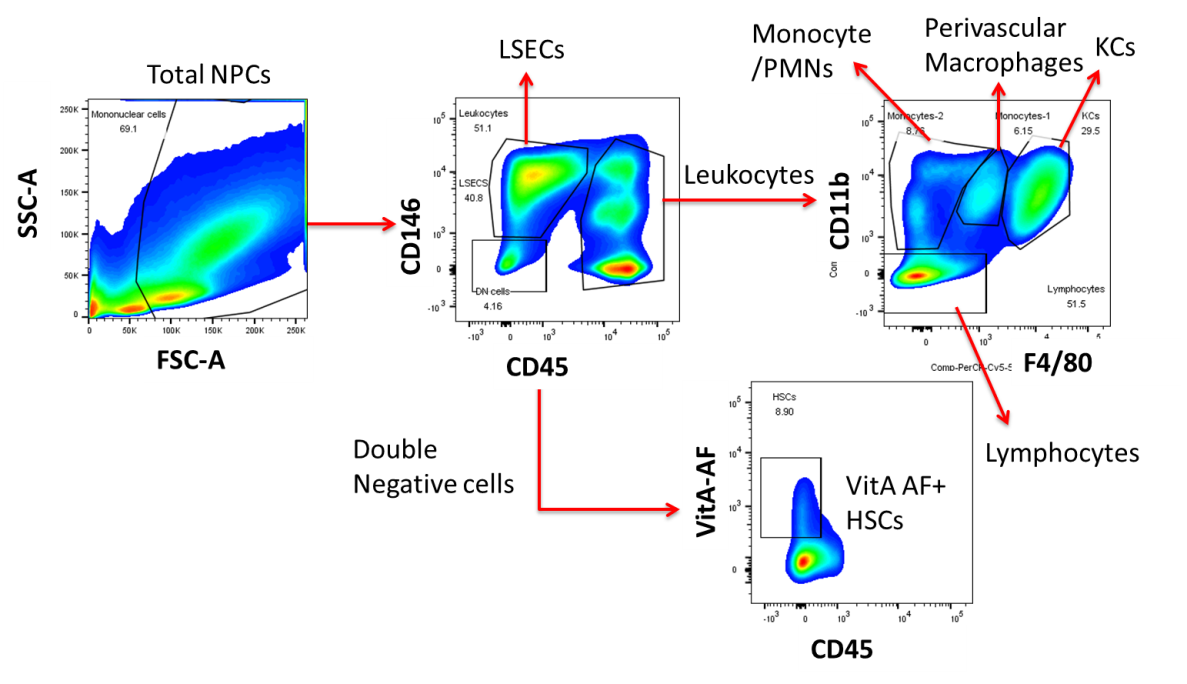
**

**Fig. S2. C**

**Figure S2. A schematic diagram of animal experiment design and strategy of liver cell isolation**

(A) Animals were administered Cy3-siRNA/HKP (+H) complex 24 hours prior to the experiment. Animals were sacrificed at 2, 4, 24 and 48 hours after final siRNA administration. Baseline samples (animals without siRNA treatment) served as a control group and were sacrificed independently. (B) Mouse liver cells were isolated by *in situ* collagenase digestion. Hepatocytes were separated by low-speed centrifugation. Total NPCs were enriched by 25%/70% Percoll gradient and distinguished by cell specific surface marker with flow-cytometry. (C) The isolated NPCs were stained with fluorochrome-conjugated antibody to discriminate each cell subpopulation. A representative flow-cytometry plot is shown to demonstrate the gating strategy of indicated cell subpopulations.


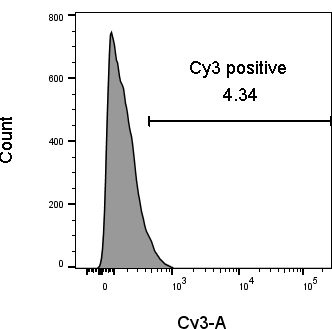

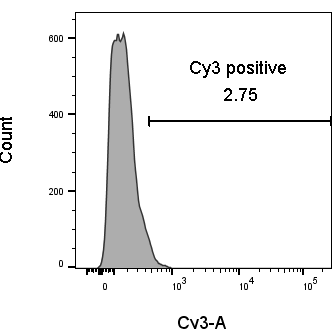

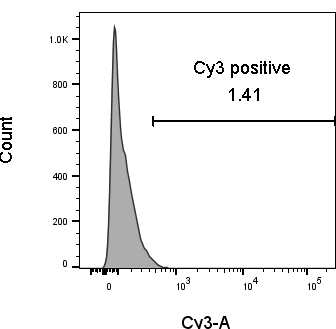


**Baseline 2H 4H**

**(A)**

**24H 48H**


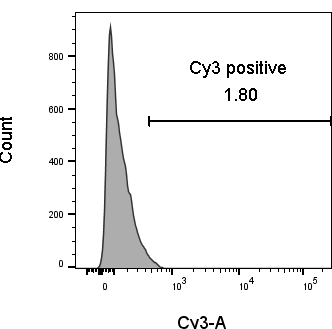

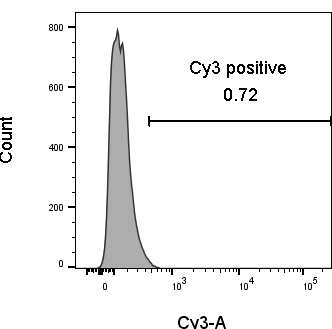

**(B)**

**Figure S3. Cy3-siRNA distribution in Hepatocytes**

(A) Representative flow-cytometry plot of Cy3-fluorescent intensity in hepatocytes. (B) Line chart of time versus Cy3-siRNA positive cell frequency analysis in hepatocytes.

In the flow analysis, the same threshold was set for fluorescence detection for different cell populations. Depending on the different cells studied, their Cy3 baseline expressions vary. Therefore, approximately 5% of false positive cells are used as a cut-off to determine how many positive cells there are in Supplemental Materials Figs S3-S9.


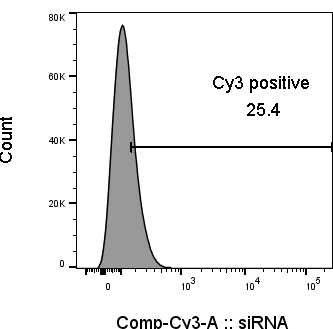

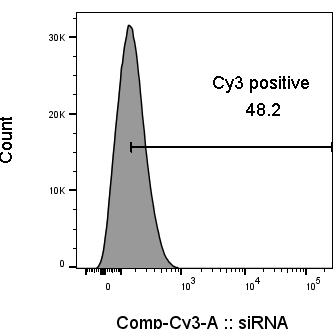


**Baseline 2H 4H**

**(A)**


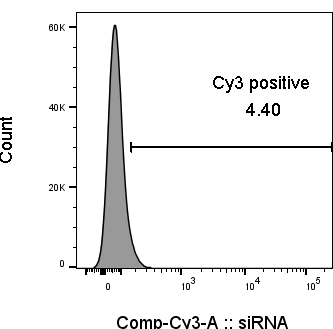


**24H 48H**


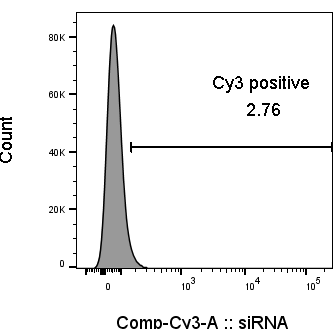

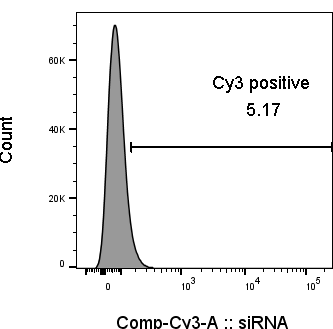

**(B)**

**Figure S4. Cy3-siRNA distribution in LSECs**

(A) Representative flow-cytometry plot of Cy3-fluorescent intensity in LSECs. (B) Line chart of time versus Cy3-siRNA positive cell frequency analysis in LSECs.

**Baseline 2H 4H**

**(A)**


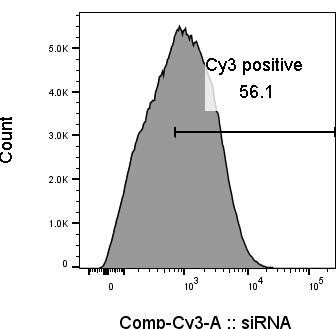

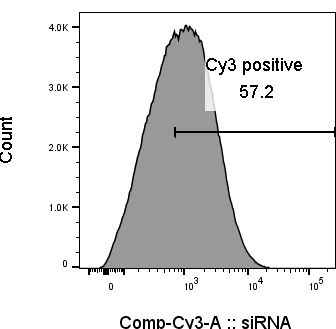

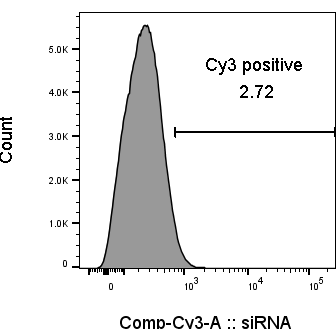


**24H 48H**


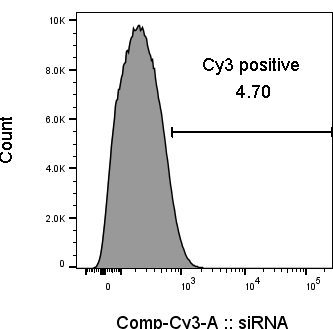

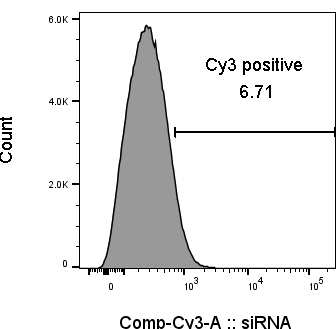

**(B)**

**Figure S5. Cy3-siRNA distribution in KCs**

(A) Representative flow-cytometry plot of Cy3-fluorescent intensity in KCs. (B) Line chart of time versus Cy3-siRNA positive cell frequency analysis in KCs.

**Baseline 2H 4H**

**(A)**


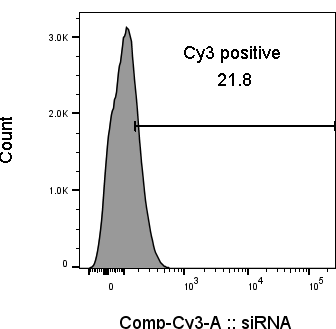

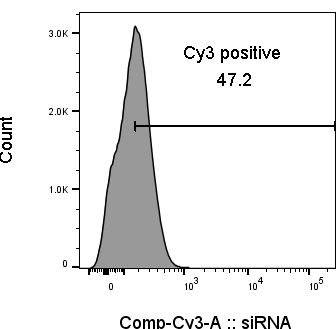

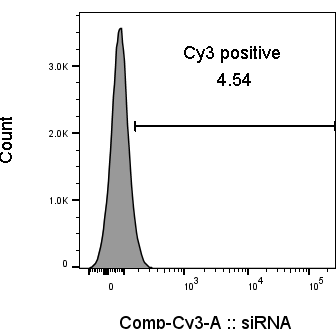


**24H 48H**


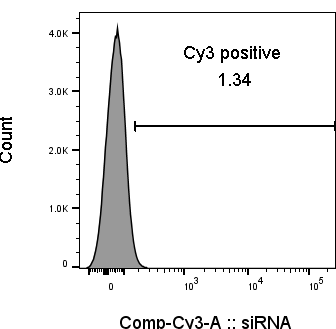

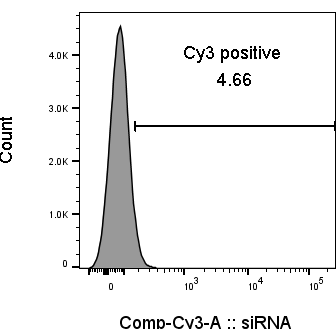

**(B)**

**Figure S6. Cy3-siRNA distribution in PVMs**

(A) Representative flow-cytometry plot of Cy3-fluorescent intensity in PVMs. (B) Line chart of time versus Cy3-siRNA positive cell frequency analysis in PVMs.

**Baseline 2H 4H**

**(A)**


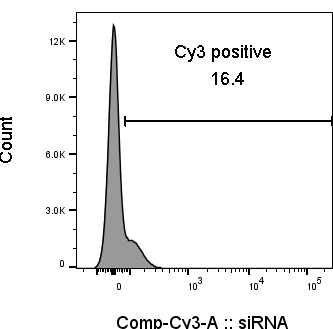

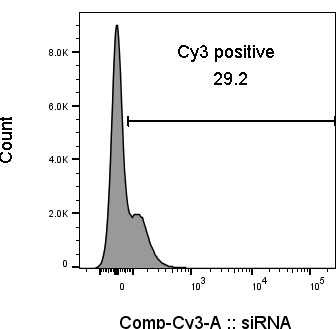

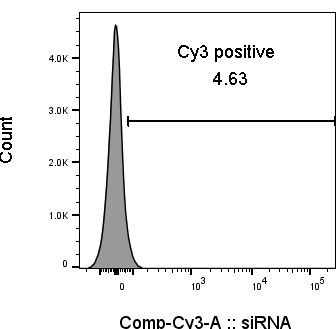


**24H 48H**


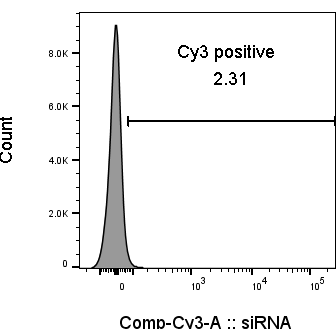

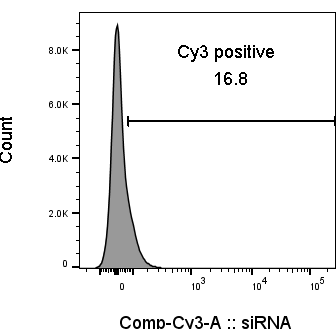

**(B)**

**Figure S7. Cy3-siRNA distribution in monocytes/PMNs**

(A) Representative flow-cytometry plot of Cy3-fluorescent intensity in monocytes/PMNs. (B) Line chart of time versus Cy3-siRNA positive cell frequency analysis in monocytes/PMNs.

**Baseline 2H 4H**

**(A)**


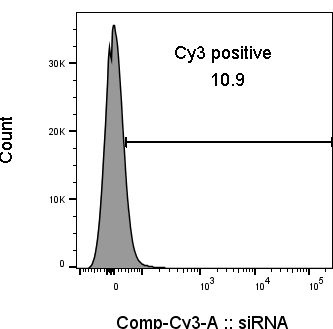

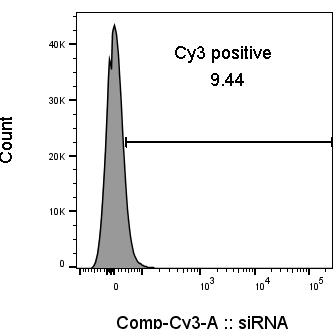

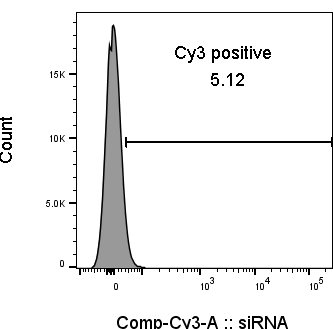


**24H 48H**


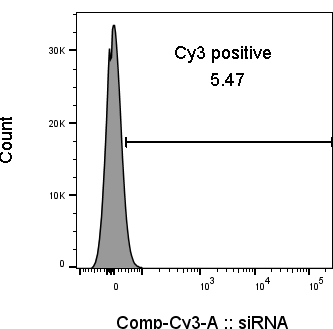

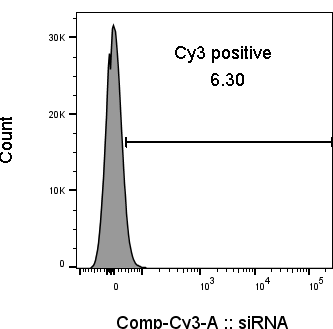

**(B)**

**Figure S8. Cy3-siRNA distribution in lymphoid cells**

(A) Representative flow-cytometry plot of Cy3-fluorescent intensity in lymphoid cells. (B) Line chart of time versus Cy3-siRNA positive cell frequency analysis in lymphoid cells.

**Baseline 2H 4H**

**(A)**


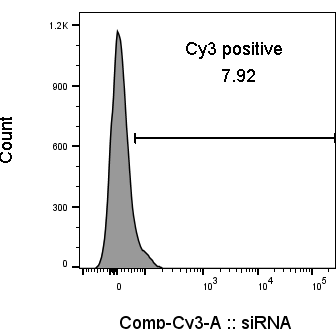

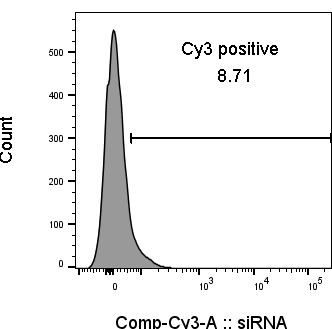

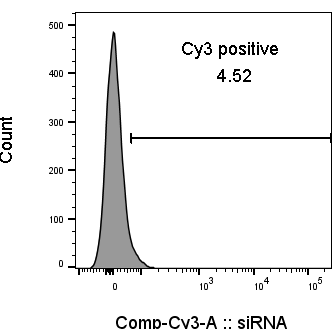


**24H 48H**


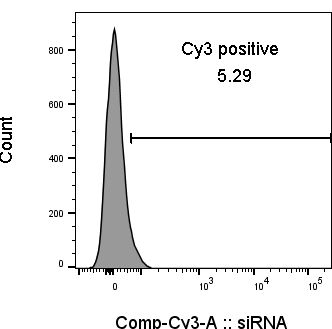

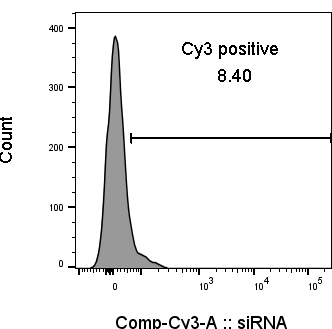

**(B)**

**Figure S9. Cy3-siRNA distribution in HSCs**

(A) Representative flow-cytometry plot of Cy3-fluorescent intensity in HSCs. (B) Line chart of time versus Cy3-siRNA positive cell frequency analysis in HSCs.

**(A)**

**
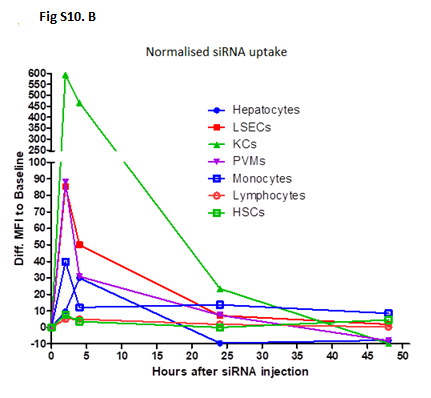
**

**Figure S10. B. Overview of Cy3-siRNA uptake by hepatic cell subpopulations**

(A) An overlaid line chart of time versus Cy3-siRNA positive cell frequency analysis in all hepatic cell subsets. (B) An overlaid line chart of time versus Cy3 fluorescent intensity in all hepatic cell subsets. The Y-axis indicates the difference between the Cy3 fluorescent intensity of baseline samples average and each 2–48-hour samples. The value represents the quantity of net Cy3 absorbance of each sample.

| Cell type | **Discriminate strategy** |
| --- | --- |
| Liver sinusoidal endothelial cells (LSECs) | CD45^+/-^CD146^+^ |
| Kupffer cells (KCs) | CD45^+^CD11b^+^F480^hi^ |
| Perivascular macrophages (PVMs) | CD45^+^CD11b^+^F480^int^ |
| Monocytes/Polymorphonuclear cells (PMNs) | CD45^+^CD11b^+^F480^-^ |
| Lymphoid cells (Mixtures of T, B, NK, and NKT cells) | CD45^+^CD11b^-^F480^-^ |
| Hepatic stellate cells (HSCs) | CD45^-^ VitA autofluorescent^+^ |
| Hepatocytes | High FSC/HSC |

**Supplementary Table.1 The discriminate strategy of each hepatic cell subpopulations on flow-cytometry**

| **Group** | **Average hepatocyte count** | **Average NPC count** | **Number** |
| --- | --- | --- | --- |
| **Baseline** | 9.3 * 10^7^ | 8.7* 10^6^ | 4 |
| **2H** | 8.4 * 10^7^ | 9.0* 10^6^ | 2 |
| **4H** | 8.6 * 10^7^ | 9.9* 10^6^ | 4 |
| **24H** | 9.4 * 10^7^ | 10.3* 10^6^ | 4 |
| **48H** | 9.8 * 10^7^ | 9.9* 10^6^ | 4 |

**Supplementary Table 2. Average isolated liver cell count of each group**

| **Cell type/group** | **Baseline** | **2H** | **4H** | **24H** | **48H** |
| --- | --- | --- | --- | --- | --- |
| **Hepatocytes** | 20075 | 18640 | 19640 | 18846 | 18678 |
| **LSECs** | 927500 | 1145000 | 1622500 | 967750 | 1057400 |
| **KCs** | 254161 | 312861 | 446500 | 302393 | 366187 |
| **PVMs** | 61117 | 103999 | 102509 | 71374 | 86979 |
| **Monocyte/PMNs** | 79637 | 169364 | 214937 | 114224 | 107224 |
| **Lymphoid cells** | 394371 | 710000 | 855750 | 587592 | 578107 |
| **HSCs** | 9555 | 12853 | 19842 | 11753 | 11374 |

**Supplementary Table 3. Average analyzed cell count on flow cytometry**

| **Cell type** | **% Cy3-siRNA positive** | **Mean fluorescent intensity** |
| --- | --- | --- |
| **Hepatocytes** | **-** | **-** |
| **LSECs** | **+++** | **+++** |
| **KCs** | **++++** | **++++** |
| **PVMs** | **+++** | **+++** |
| **Monocyte/PMNs** | **++** | **++** |
| **Lymphoid cells** | **+** | **+** |
| **HSCs** | **+** | **+** |

**Supplementary Table 4. Summary of Cy3-siRNA uptake ability of each hepatic cell type**

**5. Appendix**

Material:

1. Perfusion Buffer I, 50 ml per mouse; Ca^2+^/Mg^2+^ free HBSS

2. Perfusion Buffer II (Collagenase solution), 40 ml per mouse diluted in HBSS (Gibco 14025 contains 1.26mM Ca^2+^ and 90mM Mg^2+^) plus 0.5 mg/ml collagenase (Type IV, sigma C5138)

3. Wash Buffer, 50 ml per mouse, 3%FBS in HBSS (Gibco 14025)

4. Staining buffer: 3% FBS in PBS

5. Cell isolation buffer: 0.5% BSA, 2 mM EDTA in PBS

6. Isotonic Percoll (100%)

Methods:

*Before starting, dilute isotonic percoll by PBS to 25% and 70%, then prepare 25%/70% discontinuous percoll gradient in 15-mL tube, 4mL/layer.

**(1) Setup perfusion device**

1. Pre-warm perfusion buffer I and II to 37℃ for approximately 15 min prior to beginning the perfusion

2. Prepare tubing for perfusion and set the flow rate to 5mL/min.

3. Fill perfusion tubing with perfusion buffer I.

4. IR lamp

**(2) Liver digestion**

1. Anesthetize mice with Zoletil/Xylazine solution.

2. Open skin to expose the peritoneal membrane and expose the portal vein. Make a breach on the diaphragm using scissors and expose the upper inferior vena cava.

3. Occlude upper inferior vena cava using a vein clamp.

4. Gently catheterize the portal vein by BD Angiocath IV Catheters (Cat. 591836). Remove metal needle and wait for blood flow to fill the catheter. Cut lower inferior vena cava to allow the perfusate efflux.

5. Start pumping, then carefully attach the tubing to the catheter immediately and avoid air bubble formation. If catheterization was conducted properly, catheter immobilization is not required. The liver will turn pale immediately.

6. Keep pumping buffer I for approximate 7 mins. Turn on the IR lamp to keep animal warm.

7. During perfusion with buffer I and II, swell the liver using forceps for 10 sec to occlude buffer flow from the vena cava, repeat every 50 secs. This procedure will enhance the digestion efficiency.

8. Stop pump and switch the perfusion buffer to collagenase solution. Resume the pump.

9. Perfuse liver with 40ml of collagenase buffer (approx. 7-8 min after resuming the pump).

10. Stop the pump and remove the catheter. Do not loosen the clamp on IVC before liver removal.

**(3) Single cell suspension**

1. Carefully remove the liver from mice. Transfer the liver to a tea strainer in 10-mL petri dish and pour 30mL wash buffer into the dish. Gently disperse liver cells using a 10-mL syringe. Only liver capsules remain in the strainer

2. Filter the cell suspension through 100um nylon cell strainer.

3. Collect and filter the residual cells using another 20mL wash buffer. Pool the cells with the filtrate of step 3-3.

4. Count hepatocyte amount at this step.

**(4) Crude Liver Cell Fractionation.**

1. Centrifuge the cell suspension at 50 × g for 5 min at 4C. At this speed and duration, hepatocytes and debris will pellet while most NPCs will remain in suspension.

2. For NPC isolation, transfer the supernatant to a new 50-ml tube. And proceed to section (5).

3. For hepatocytes, re-suspend hepatocyte pellet in 10mL hepatocyte wash buffer (10% FBS william’s medium E).

4. Filter hepatocyte suspension using 100um nylon mesh again, collect 0.5-1mL filtrate sufficient for flow cytometry analysis.

5. Pellet filtrate at 50 × g for 5 min, 4C. Fix cell pellet with 1mL cell fixation buffer (4%PFA) for 30 mins in dark.

6. Wash cells with 2mL cell staining buffer once.

7. Hepatocytes are re-suspended in 0.5mL cell staining buffer and are ready for FACS analysis.

**(5) NPC enrichment**

1. For the supernatant of step 2, Centrifuge the cell suspension at 50 × g for 5 min, 4C again and transfer the supernatant to a new 50-ml tube.

2. Pellet the NPC suspension at 400 × g for 10 min at 4C.

3. Re-suspend NPC pellet in 4-mL PBS and mix thoroughly by pipetting 3~5 times.

4. Layer the cell suspension on the top of previously prepared 25%/70% percoll gradient.

5. Centrifuge at 1350xg, 30 mins, without a break.

6. Collect 25%/70% interphase and wash with 15mL PBS, centrifuge at 400xg, 10 mins, 4C.

7. Re-suspend cell pellet using 0.5mL staining buffer, adjust cell concentration to 2*10^7 cells/mL (2*10^6/0.1mL). 1 Rxn= 0.1 mL cell suspension.

**(6) Flow cytometry staining**

1. Add 2uL BD mouse Fc block per 0.1mL cell suspension. Incubate at 4C for 15 mins.

2. During incubation, prepare staining antibody cocktail as described following.

| Fluorochrome/Tube name | Liver NPC | Isotype |
| --- | --- | --- |
| FITC (2uL) | CD146 | Isotype |
| Cy3 | Blank (siRNA) | Blank (siRNA) |
| PerCp-Cy5.5 (5uL) | F4/80 | Isotype |
| APC (5uL) | CD45 | Isotype |
| PE-Cy7 (5uL) | CD11b | Isotype |

For one Rxn, dilute antibodies in Brilliant staining buffer to total volume = 50uL

3. Aliquot cell suspension into 3 parts, add antibody mixture at 1:1 ratio to the cell suspension. Incubate cells at 4C for 30 mins in dark.

4. Wash twice with 2mL staining buffer per tube. Centrifuge at 400xg, 5 mins, 4C.

If cells are not analyzed immediately, proceed to step (7)

5. Re-suspend cells in 0.5mL staining buffer, cells are ready for flow cytometric analysis.

(7) Fixing cells for analysis

1. Re-suspend cells in 0.5mL Fluorofix buffer (Biolegend). Incubate cells at 4C for 30 mins in dark.

2. Wash with 2mL staining buffer per tube. Centrifuge at 400xg, 5 mins, 4C.

3. Re-suspend cells in 0.5mL staining buffer, cells are ready for flow cytometric analysis.

**References**

1. Carthew RW & Sontheimer EJ (2009) Origins and Mechanisms of miRNAs and siRNAs. *Cell* 136(4):642-655.

2. Wittrup A & Lieberman J (2015) Knocking down disease: a progress report on siRNA therapeutics. *Nat Rev Genet* 16(9):543-552.

3. Yu X*, et al.* (2019) Immune modulation of liver sinusoidal endothelial cells by melittin nanoparticles suppresses liver metastasis. *Nature Communications* 10(1):574.

4. Yona S*, et al.* (2013) Fate Mapping Reveals Origins and Dynamics of Monocytes and Tissue Macrophages under Homeostasis. *Immunity* 38(1):79-91.

5. Mederacke I, Dapito DH, Affò S, Uchinami H, & Schwabe RF (2015) High-yield and high-purity isolation of hepatic stellate cells from normal and fibrotic mouse livers. *Nature Protocols* 10(2):305-315.

6. Mohar I, Brempelis KJ, Murray SA, Ebrahimkhani MR, & Crispe IN (2015) Isolation of Non-parenchymal Cells from the Mouse Liver. *Methods in molecular biology (Clifton, N.J.)* 1325:3-17.

7. Charni-Natan M & Goldstein I (2020) Protocol for Primary Mouse Hepatocyte Isolation. *STAR Protocols* 1(2):100086.

8. Bale SS, Geerts S, Jindal R, & Yarmush ML (2016) Isolation and co-culture of rat parenchymal and non-parenchymal liver cells to evaluate cellular interactions and response. *Sci Rep* 6:25329-25329.

9. De Bleser PJ, Niki T, Rogiers V, & Geerts A (1997) Transforming growth factor-β gene expression in normal and fibrotic rat liver. *Journal of Hepatology* 26(4):886-893.

10. Thomson AW & Knolle PA (2010) Antigen-presenting cell function in the tolerogenic liver environment. *Nature Reviews Immunology* 10(11):753-766.
